# Engineered silica nanoparticles act as adjuvants to enhance allergic airway disease in mice

**DOI:** 10.1186/1743-8977-10-26

**Published:** 2013-07-01

**Authors:** Christina Brandenberger, Nicole L Rowley, Daven N Jackson-Humbles, Quanxuan Zhang, Lori A Bramble, Ryan P Lewandowski, James G Wagner, Weimin Chen, Barbara L Kaplan, Norbert E Kaminski, Gregory L Baker, Robert M Worden, Jack R Harkema

**Affiliations:** 1 Department of Pathobiology and Diagnostic Investigation, Michigan State University, East Lansing, USA; 2 Department of Chemistry, Michigan State University, East Lansing, USA; 3 Center for Integrative Toxicology, Michigan State University, East Lansing, USA; 4 Department of Microbiology and Molecular Genetics, Michigan State University, East Lansing, USA; 5 Department of Pharmacology and Toxicology, Michigan State University, East Lansing, USA; 6 Department of Chemical Engineering, Michigan State University, East Lansing, USA

**Keywords:** Silica nanoparticles, Adjuvant potential, Allergic airway disease, Th2/Th17 response, Murine ovalbumin model

## Abstract

**Background:**

With the increase in production and use of engineered nanoparticles (NP; ≤ 100 nm), safety concerns have risen about the potential health effects of occupational or environmental NP exposure. Results of animal toxicology studies suggest that inhalation of NP may cause pulmonary injury with subsequent acute or chronic inflammation. People with chronic respiratory diseases like asthma or allergic rhinitis may be even more susceptible to toxic effects of inhaled NP. Few studies, however, have investigated adverse effects of inhaled NP that may enhance the development of allergic airway disease.

**Methods:**

We investigated the potential of polyethylene glycol coated amorphous silica NP (SNP; 90 nm diameter) to promote allergic airway disease when co-exposed during sensitization with an allergen. BALB/c mice were sensitized by intranasal instillation with 0.02% ovalbumin (OVA; allergen) or saline (control), and co-exposed to 0, 10, 100, or 400 μg of SNP. OVA-sensitized mice were then challenged intranasally with 0.5% OVA 14 and 15 days after sensitization, and all animals were sacrificed a day after the last OVA challenge. Blood and bronchoalveolar lavage fluid (BALF) were collected, and pulmonary tissue was processed for histopathology and biochemical and molecular analyses.

**Results:**

Co-exposure to SNP during OVA sensitization caused a dose-dependent enhancement of allergic airway disease upon challenge with OVA alone. This adjuvant-like effect was manifested by significantly greater OVA-specific serum IgE, airway eosinophil infiltration, mucous cell metaplasia, and Th2 and Th17 cytokine gene and protein expression, as compared to mice that were sensitized to OVA without SNP. In saline controls, SNP exposure did cause a moderate increase in airway neutrophils at the highest doses.

**Conclusions:**

These results suggest that airway exposure to engineered SNP could enhance allergen sensitization and foster greater manifestation of allergic airway disease upon secondary allergen exposures. Whereas SNP caused innate immune responses at high doses in non-allergic mice, the adjuvant effects of SNP were found at lower doses in allergic mice and were Th2/Th17 related. In conclusion, these findings in mice suggest that individuals exposed to SNP might be more prone to manifest allergic airway disease, due to adjuvant-like properties of SNP.

## Background

Engineered nanoparticles (NP) have unique and desirable functional properties, due to their extremely small size (≤ 100 nm). As a result, NP have an enormous economic potential and are rapidly being introduced into commercial products, such as textiles, cosmetics and food packaging [1]. Recent reviews [2,3], however, have presented compelling evidence that occupational exposure during the production of NP might have significant health risks. Due to their small size and potential for airborne dispersion, inhalation of NP may be a plausible route of human exposure in the workplace. Under certain conditions, inhalation of NP could potentially exacerbate or contribute to the onset of common respiratory diseases, such as chronic bronchitis, asthma or allergic rhinitis [3,4].

Asthma and other allergic airway diseases are a worldwide health problem. In the United States approximately 26 million people suffer from asthma (National Health Interview Survey, National Center for Health Statistics, CDC, 1980–2009). Asthma is characterized by reversible airway obstruction, airway hyperresponsiveness (AHR), increased production of immunoglobulin isotype IgE, airway inflammatory cell infiltrates of eosinophils and CD4^+^ T helper type 2 (Th2) lymphocytes, mucus hypersecretion, and airway remodeling (e.g., epithelial mucous cell metaplasia, intramural interstitial fibrosis).

Various factors have been recognized to exacerbate asthma such as indoor and outdoor allergens, tobacco smoke and air pollution [5]. In urban air pollution, particulate matter (PM) is a well-recognized risk factor, causing exacerbation of asthmatic symptoms (e.g., dyspnea, airway constriction, airway mucus hypersecretion). In addition, results of recent animal toxicology studies have demonstrated that exposure to intranasally aspirated ultrafine PM (UFP; < 100 nm) during sensitization with ovalbumin (OVA), a commonly used experimental allergen, can enhance the development and severity of allergic airway disease in mice [6,7]. UFP acted as adjuvants to boost the secondary immune response upon subsequent OVA challenge. In these studies, the adjuvant potential of UFP was closely associated with their oxidant potential [7]. These laboratory findings 1) suggest a plausible biological mechanism for epidemiological reports that PM exposure caused enhancement of morbidity in humans with allergic airway disease [8] and 2) provide a reproducible *in vivo* murine model to test the potentially adverse adjuvant effects of other NP, such as engineered NP that have distinctive physical and chemical characteristics.

In the present study, we used an OVA-induced murine model of asthma to test the hypothesis that engineered amorphous silica nanoparticles (SNP) may act as inhaled adjuvants to enhance allergic airway disease. SNP are used as additives to cosmetics, drugs, printer toners, varnishes and food [9]. It is well known that chronic inhalation exposure of coarse-sized (2.5 to 10 μm), crystalline silica particles can lead to a debilitating fibrotic condition known as pulmonary silicosis [10]. In contrast, synthetic amorphous silica particles are thought to be much less toxic to the lung. Inhalation of engineered amorphous silica causes only minimal and transient pulmonary inflammation in laboratory rodents [11,12] and no fibrosis of the lungs [13,14] as compared to crystalline silica particles. Few toxicology studies have been conducted to examine the adverse effects of inhaled amorphous SNP and, to the best of our knowledge, no studies have been designed to investigate the potential of these NP to act as adjuvants to enhance the development or exacerbation of allergic airway disease.

**Figure 1:**
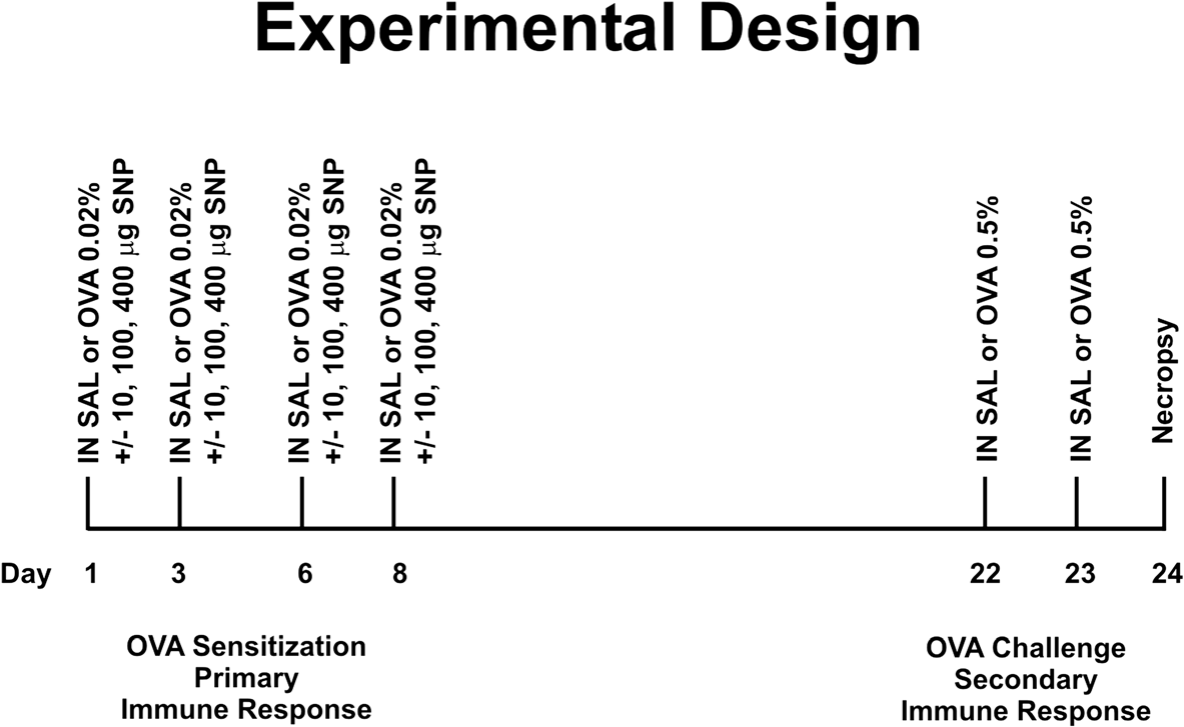
**Study design and exposure scheme. **Mice were sensitized intranasally (IN) on days 1, 3, 6 and 8 with 0.02% OVA or saline (SAL). SNP were co-administered at intranasal doses of 0, 10, 100, or 400 μg with OVA or saline. On days 22 and 23, OVA-mice were challenged intranasally with 0.5% OVA and SAL-mice with saline solution. Animals were sacrificed on day 24, 24 hours after the last intranasal challenge.

The adjuvant potential of SNP was determined by assessing the magnitude of OVA-induced histopathological and immunological responses in the lung of mice, which were intranasally instilled with 0, 10, 100 or 400 μg SNP, at four distinct times, along with OVA (i.e., antigen sensitization with or without SNP) and 14 days prior to subsequent OVA challenge. Amorphous SNP had a hydrodynamic diameter of 90 nm (Table 1) and were coated with a polyethylene glycol (PEG) shell to prevent them from agglomeration [15]. A scheme of the study design is presented in Figure 1.

**Table 1:** **Grafting amount and size of SNP after different steps of synthesis**: plain SNP (SNP) to amine-modified SNP (aSNP), to alkyne-modified (aaSNP) and to final PEG-coated SNP

**Sample**	**Weight Loss (%)**^**a**^	**Grafting amount (t, mmol/g)**^**a**^	**Grafting density (group/nm**^**2**^**)**^**a**^	**Size (nm)**^**b**^
**plain SNP**	1.7^g^	1.9^h^	8.1^h^	30^c^
**aSNP**	3.6	0.63	2.7	103^d^
**aaSNP**	2.4	0.29	1.2	126^e^
**PEG-coated SNP**	3.6	0.15	0.7	90^c^

^a^Weight loss, grafting amount and density were calculated for each modification step, not for total modification. ^b ^Size were measured by dynamic light scattering as mono-distribution after sonication for 30-60 min. ^c ^Size was measured in H_2_O. ^d ^Size was measured in dimethylformamide triethylamine (DMF-TEA) (3:1). ^e ^Size was measured in DMF. ^g ^Weight loss of water from surface of bare silica particle. ^h^ Grafting amount and density of Si-OH available for modification on the surface calculated from water loss.

## Results

### OVA induced allergic airway disease

**Figure 2:**
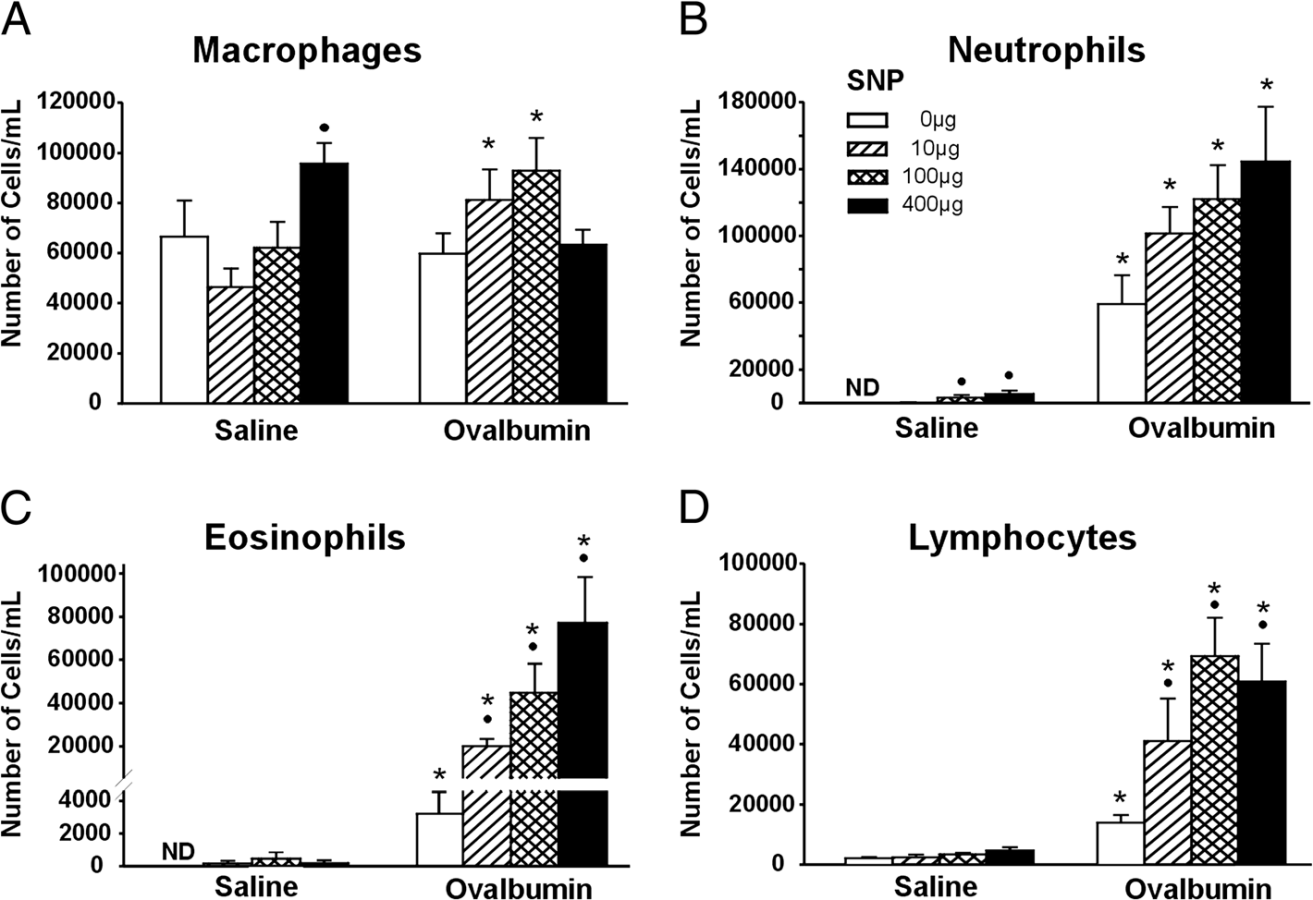
**Differential BALF cell counts. **Differential counts of macrophages (**A**), neutrophils (**B**), eosinophils (**C**) and lymphocytes (**D**) were assessed in all study groups. •: Significant changes (p < 0.05) when compared to non-SNP exposed animals, *: significant changes when compared to SAL-mice.

Animals that were treated with OVA without SNP (OVA-mice) had a significant (p ≤ 0.05) 2-fold increase in total BALF cells compared to saline-treated control mice (SAL-mice; 68,750 ± 14,372 and 135,833 ± 21,337 for SAL- and OVA-mice, respectively). This increase in total cells was due to a significant increase in neutrophils, eosinophils and lymphocytes (Figure 2). Whereas no eosinophils or neutrophils were detected in the BALF of SAL-mice, 2.2% and 39% of BALF cells were eosinophils and neutrophils in the OVA-mice (Figure 2B and C). In addition, lymphocytes were 7-fold greater in OVA-mice, compared to SAL-mice, and accounted for 11% of the BALF cells in these animals (Figure 2D).

**Figure 3:**
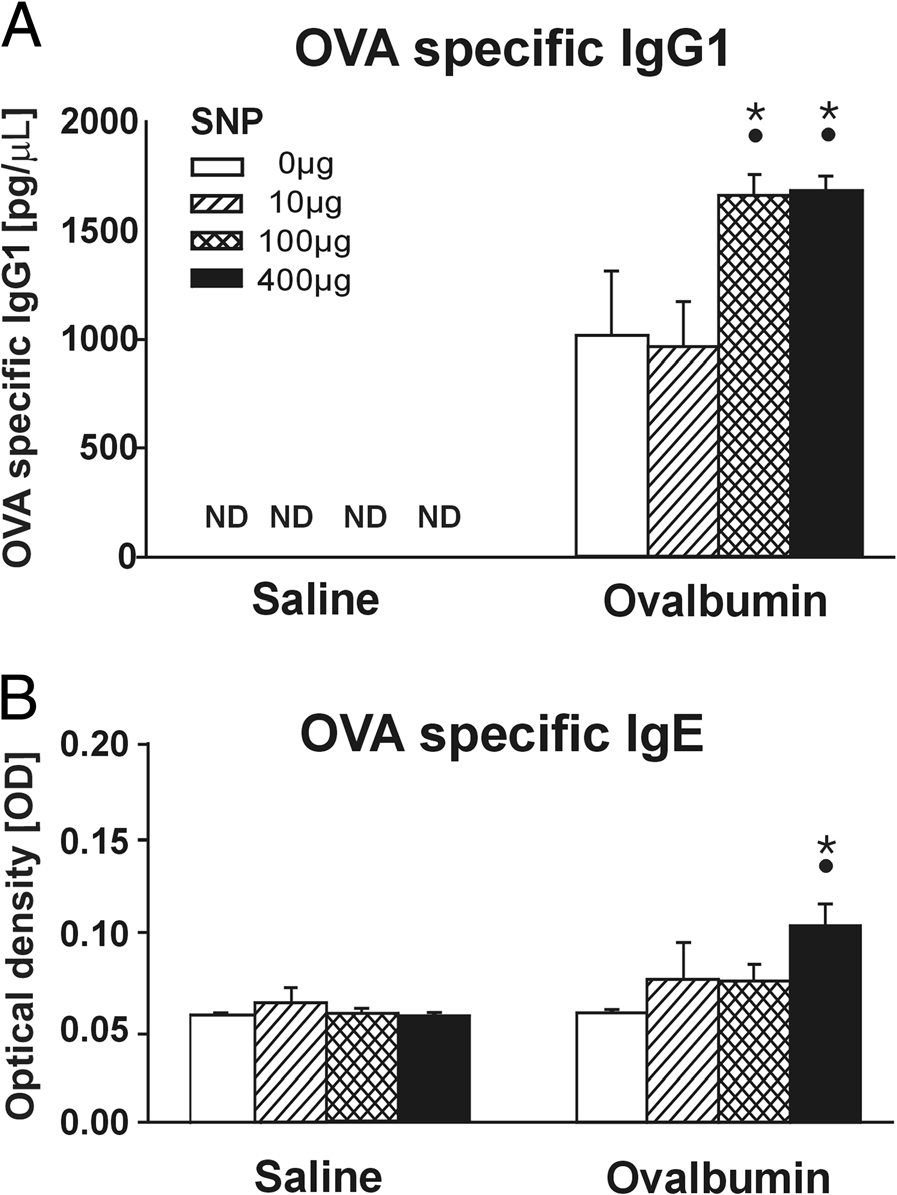
**OVA specific IgE and IgG1 expression. **Serum levels of OVA specific IgG1 (**A**) are detected by an ELISA and presented as total serum concentration. OVA-specific IgE levels (**B**) are measured as optical density (OD). •: Significant changes (p < 0.05) when compared to non-SNP exposed animals, *: significant changes when compared to non-allergic controls.

OVA sensitization and challenge also caused expression of OVA-specific IgG1 antibodies in serum, another characteristic feature of a type II hypersensitivity immune response to this foreign antigen (Figure 3A). However, no elevation of OVA-specific serum IgE antibodies was detected in OVA-mice (Figure 3B).

**Figure 4:**
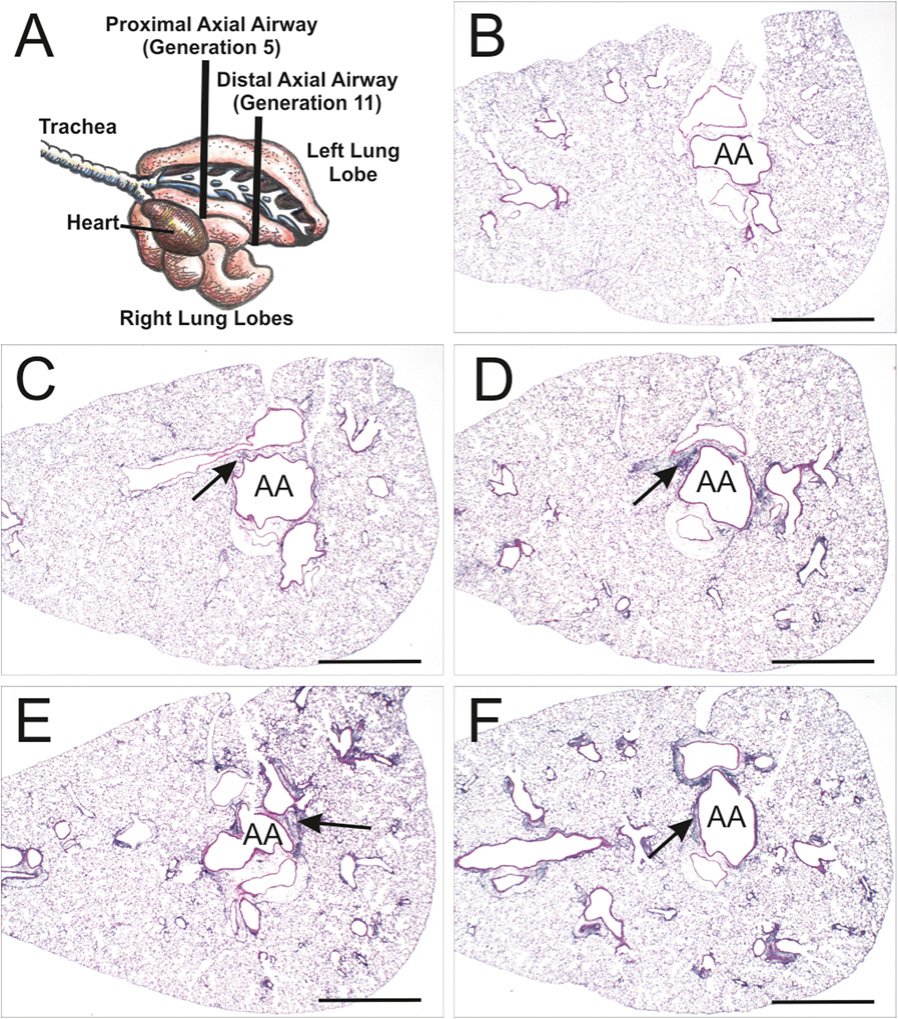
**Pulmonary histopathology. **A diagram illustrates the locations of transverse tissue sections taken from the left lung lobe for microscopic examination (**A**). Light photomicrographs of representative lung sections taken at the level of the fifth axial airway (AA) generation and stained with hematoxylin and eosin (**B**-**F**). Representative light photomicrographs of a control animal (SAL-mouse; **B**), OVA-mouse (**C**) and SNP/OVA-mice with increasing SNP exposure doses (**D**-**F**) illustrating peribronchiolar and perivascular mixed inflammatory cell infiltration in OVA- and SNP/OVA-mice (arrows). Greater airway-associated inflammation is present in SNP/OVA-mice exposed to concentrations of 100 and 400 μg SNP (E and F, respectively) compared to the SAL- and OVA-mice. Scale bars = 1 mm.

**Figure 5:**
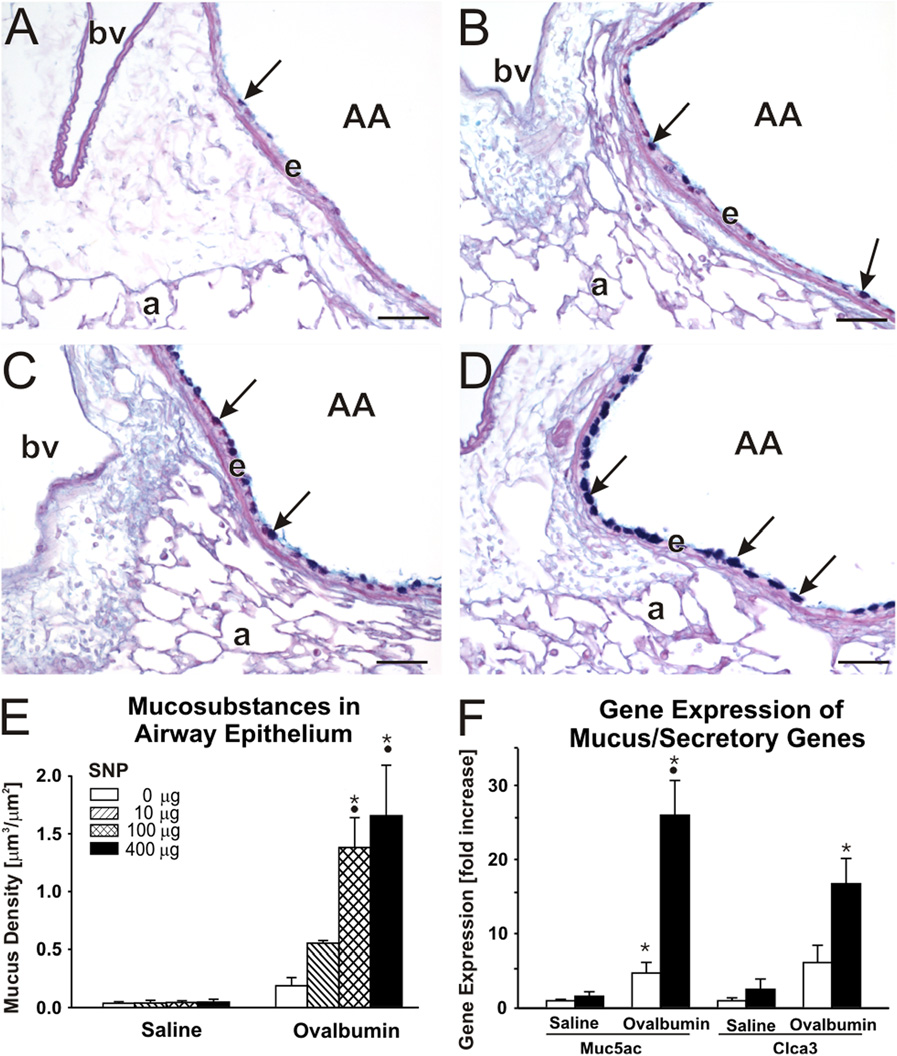
**Airway epithelial mucus production. **Increase in airway epithelial mucus, as a feature of allergic airway disease, was analyzed on lung tissue at the 5th generation of the intraepithelial AB/PAS stained mucosubstances (arrows) in SNP/OVA-mice with increasing SNP exposure dose are shown in figure **A** (0 μg), **B** (10 μg), **C **(100 μg) and **D** (400 μg); AA = axial airway, e = airway epithelium, a = alveoli, bv = blood vessel. Morphometric measurement of intraepithelial AB/PAS mucosubstances are further shown in **E** and changes in *Muc5ac* and *Clca3* gene expression in **F**. •: Significant changes (p < 0.05) when compared to non-SNP exposed animals, *: significant changes when compared to non-allergic controls.

Histopathologically, there was a conspicuous peribronchiolar and perivascular mixed inflammatory cell influx, that was principally located in the proximal aspect of the lung lobe (G5 tissue section; Figure 4A-C). This airway inflammatory response to OVA was most prominent around large diameter pre-terminal bronchioles with occasional extension to the more distal terminal bronchioles. OVA treatment also caused airway epithelial remodeling characterized by mucous cell metaplasia, as identified with Alcian Blue (pH 2.5)/Periodic Acid–Schiff (AB/PAS) staining for intraepithelial neutral and acidic mucosubstances in pulmonary bronchiolar epithelium (Figure 5). Normally the bronchiolar epithelium of mice has no or very few such secretory cells. This metaplastic epithelial response was restricted primarily to large-diameter pre-terminal bronchioles in the proximal G5 lung section of OVA-treated mice (Figure 5A). Morphometrically, the volume density of AB/PAS-stained mucosubstances in the proximal (G5) axial airway of the left lung lobe did not reveal any significant difference between SAL- and OVA-mice (Figure 5E). However, lungs of OVA-mice also had an induced gene expression of mucin 5 AC (*Muc5ac*) and chloride channel calcium activated 3 (*Clca3, Gob5*), both of which are associated with goblet cell hyperplasia as well as airway mucus secretion in allergic airway disease [16]. There was a significant 4.6-fold increase in *Muc5ac* gene expression in OVA-mice relative to SAL-mice (Figure 5F).

### Adjuvant effects of SNP

The exposure of SNP during OVA sensitization (SNP/OVA-mice) resulted in an exacerbation of allergic airway disease after OVA challenge, hence implying an adjuvant effect of SNP in the development of asthma. The adjuvant effect observed in SNP/OVA-mice increased with SNP dose (0, 10, 100, 400 μg SNP/animal).

Total BALF cells were significantly increased up to 2.5-fold in SNP/OVA-mice compared to OVA-mice (135,833 ± 21,337, 257,917 ± 48,958, 323,333 ± 40,284 and 345,833 ± 66,476 for OVA- and SNP/OVA-mice with SNP doses of 10, 100 and 400 μg, respectively). The adjuvant effect was consistently demonstrated in the number of BALF eosinophils. Significant, dose-dependent increases in lavaged eosinophils were present in SNP/OVA-mice compared to OVA-mice (11-, 14- and 25-fold increases for 10, 100 and 400 μg SNP/OVA co-exposures respectively; Figure 2C). A significant increase was also observed for BALF lymphocytes in SNP/OVA-mice relative to OVA-mice (2.9-, 5.0- and 4.4-fold for 10, 100 and 400 μg SNP/OVA, respectively; Figure 2D). No adjuvant-related increases were found for macrophages and neutrophils (Figure 2A and B).

Serum OVA-specific IgG1 antibody levels (Figure 3A) were significantly elevated (1.6-fold) in 100 and 400 μg SNP/OVA-mice compared to OVA-mice. A significant increase in OVA-specific IgE antibodies levels was also detected in 400 μg SNP/OVA-mice (Figure 3B), which was not present in any other exposure group. A systemic allergic response was observed only in mice exposed to SNP during OVA sensitization at the highest SNP dose.

**Figure 6:**
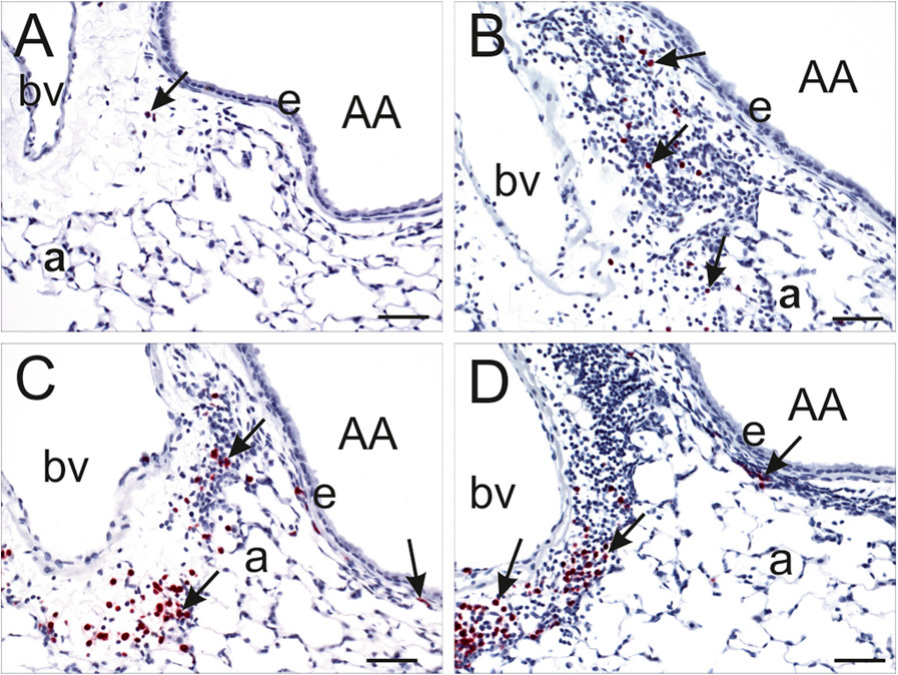
**Immunohistochemistry of airway-associated eosinophils.** Light photomicrographs of peri-bronchiolar and –vascular interstitium surrounding the proximal axial airway (AA) at generation 5. Tissues were immunohistochemically stained for eosinophils (murine-specific anti-major basic protein antibody; red chromagen; arrows) and counterstained with hematoxylin. OVA-induced inflammatory cell infiltrate composed of eosinophils and mononuclear cells (lymphocytes and plasma cells) is dose-dependently enhanced by SNP. Figures **A**-**D** are taken from OVA-treated mice that were co-exposed to 0 (saline control), 10, 100 and 400 μg SNP, respectively. bv: blood vessel; e: airway epithelium; a: alveolus; Scale bars = 50 μm.

Microscopically there was a SNP dose dependent, peribronchiolar and perivascular mixed inflammatory cell influx in SNP/OVA-mice, that was principally located in the proximal lung lobe (G5 tissue section), with some extension into the distal lung lobe (Figure 4C-F). The inflammatory cell influx was composed mainly of lymphocytes and lesser numbers of eosinophils (Figure 6). This airway inflammatory response to OVA and SNP/OVA was most prominent around large diameter pre-terminal bronchioles with extension to the more distal terminal bronchioles. Inflammatory cell infiltration was also evident in the interstitial tissues surrounding pulmonary arteries adjacent bronchiolar airways and pulmonary veins embedded in the alveolar parenchyma.

Intracellular mucosubstances in epithelial cells lining the proximal axial airway (G5) increased with increasing SNP dose in the SNP/OVA-mice (Figure 5A-E). There was a significant 6 to 7-fold increase in 100 and 400 μg SNP/OVA-mice compared to OVA-mice. Gene expression analysis of *Muc5ac* and *Clca3* revealed 5.5-fold (p < 0.001) overexpression of *Muc5ac* in 400 μg SNP/OVA as compared to OVA-mice (Figure 5F).

### SNP co-sensitization induces a Th2/Th17 cytokine response in OVA-mice

To investigate the mechanisms of SNP adjuvancy in the development of allergic airway disease, a gene expression array of lung tissue and BALF cytokine expression analysis were performed. For gene expression analysis, 96 genes were chosen as part of different gene clusters (i.e. chemokines, cytokines and other immune responsive genes, mucus/surfactant production and secretion, growth factors and cell cycle, oxidative stress and redox response as well as transcription factors). A detailed list of all genes is available in the supplemental information (Additional file 1: Table S1) as well as an expression heat map of all analyzed genes (Additional file 1: Figure S5). For BALF cytokine analysis, a panel of acute phase, Th1, Th2 and Th17 cytokines and chemokines was used, including interleukin-1β (IL1β), IL2, IL4, IL5, IL6, IL 13 and IL17, keratinocyte chemoattractant (KC; CXCL1), macrophage inflammatory protein 1α (MIP-1α; CXCL3), monocyte chemoattractant protein 1 (MCP-1; CCL2), tumor necrosis factor α (TNFα), and interferon γ (IFNγ).

**Figure 7:**
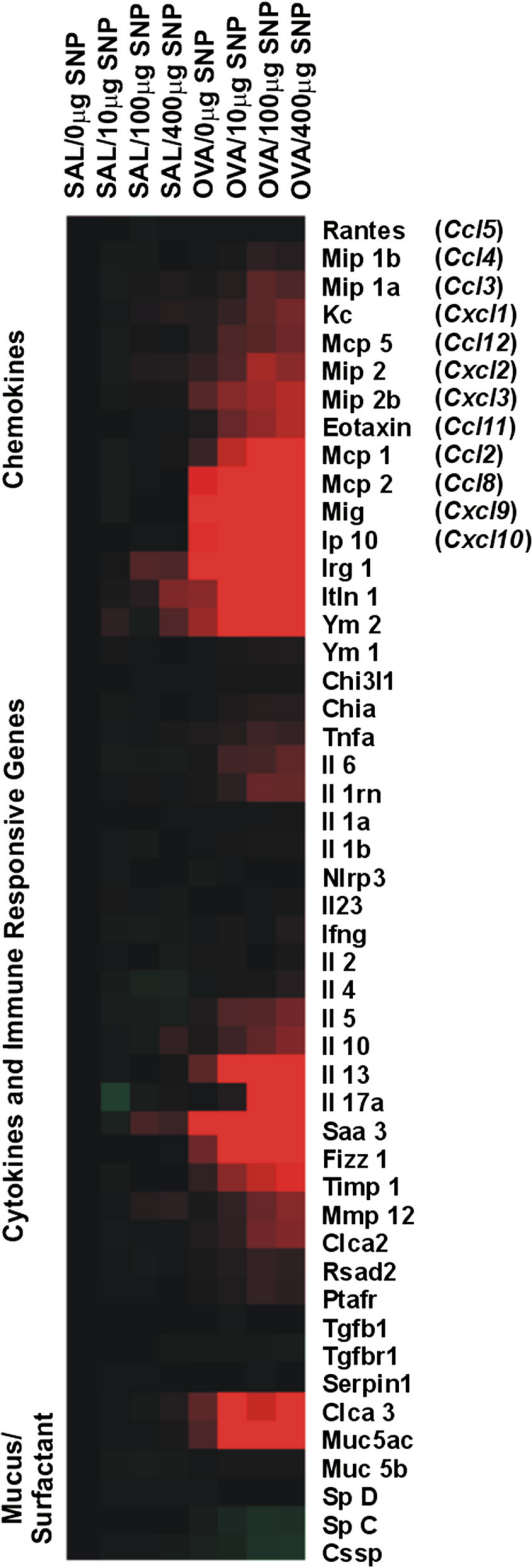
**Heat map of gene expression array.** Gene expression in lung tissue was analyzed with a 96-gene array. Relative increased gene expression towards control is indicated in red (≥2 fold expression) and relative decreased gene expression in green (≤2 fold). Black labels indicate no differences in gene expression. Increased gene expression in allergic and SNP exposed animals was found for various cytokines, chemokines and immune responsive genes as well as secretory mucus/surfactant genes. No changes greater than 2-fold were found for oxidative stress response, growth factors and different transcription factors (Additional file 1: Figure S5).

Gene expression analysis was first performed with pooled cDNA of all individuals within a study group (n = 6 animals/group). Relevant changes in gene expression levels (>2 fold of SAL-mice; calculated with relative ΔΔC_t_ method) in OVA- and SNP/OVA-mice were found in gene clusters of chemokines, cytokines and inflammation as well as of mucus/surfactant production and secretion. These results are presented as a heat map in Figure 7. No elevated changes were found in other gene clusters, such as those for cell cycle and growth factors, oxidative stress or transcription factors (Additional file 1: Figure S5). To confirm findings observed in the aforementioned gene arrays, a full qPCR analysis on non-pooled, individual samples of SAL-mice, OVA-mice, 400 μg SNP-mice and 400 μg SNP/OVA-mice was performed on genes with >2 fold change compared to control.

**Figure 8:**
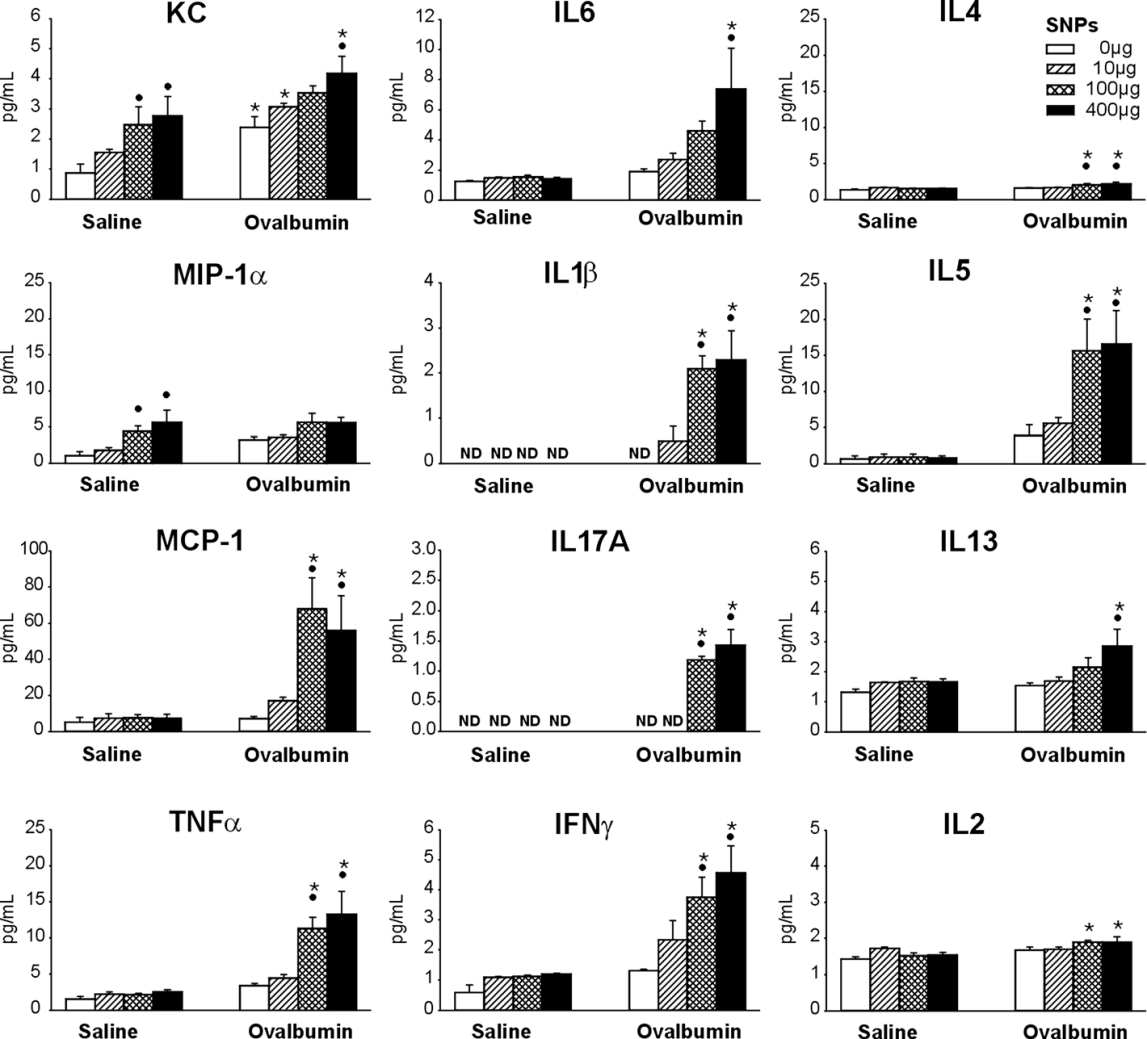
**Expression of BALF cytokines and chemokines.** Different BALF cytokine expression profiles were analyzed by an ELISA as for KC, MIP-1α, MCP-1, TNFα, IFNγ and IL1β, IL2, IL4, IL5, IL6, IL13, IL17A. •: Significant changes (p < 0.05) when compared to non- SNP exposed animals, *: significant changes when compared to non-allergic controls.

The results and evaluation of the selected genes are presented in Table 2. SNP/OVA-mice showed a significant elevation in gene expression of Th2 cytokines (*Il4, Il5, Il13*) and Th17 cytokines and related genes (*Il17a*; *Il6*; serum amyloid A3, *Saa3*) compared to OVA-mice. Gene expression of monocyte and eosinophil chemokines such as *Mcp-1* (*Ccl2*), *Mcp-2* (*Ccl8*), inflammatory protein (*Ip10, Cxcl10*) and *eotaxin* (*Ccl11*) were further significantly increased in OVA- and SNP/OVA-mice, as well as genes of proteins that are associated with tissue remodeling such as tissue inhibitor of metalloproteinase 1 (*Timp1*) and resistin-like molecule alpha (*Retnla*, *Fizz1*). TIMP1 antagonizes tissue remodeling induced by metalloproteinase 9 [17] and FIZZ1 is an early biomarker for tissue remodeling in asthma [18]. Though all these genes were significantly elevated in both OVA- and SNP/OVA-mice compared to SAL-mice, the response was always greater in SNP/OVA-mice. This was also the case for regulatory T cell (T-reg) cytokines and transcription factor (*Il10*; forkhead protein P3, *Foxp3*). The adjuvant Th2/Th17 cytokine response was also confirmed in BALF analysis (Figure 8). Acute phase cytokines (TNFα, IL1β), Th2 cytokines (IL4, IL5, IL13) and Th17 cytokines (IL17A, IL6) were significantly increased in SNP/OVA-mice at SNP doses of 400 μg and IL4, IL5 and IL17A already in SNP/OVA-mice exposed at 100 μg SNP. OVA exposure, without SNP, did not induce an increase in BALF cytokines, with the only exception of KC, a neutrophil activation chemokine, which was elevated in both OVA- and SNP/OVA-mice. Furthermore a significant increase in Th1 cytokine IFNγ was observed, as evidenced by increased gene expression in lung tissue of OVA- and SNP/OVA-mice and increased cytokine levels in BALF of SNP/OVA-mice. However, there was no adjuvant increase for the Th1 cytokine IL2.

**Table 2:** Detailed gene expression analysis of responsive genes

**Gene symbol**	**Gene alias**	**Gene function**	**Saline/0 μg NP**	**Saline/400 μg NP**	**OVA/0 μg NP**	**OVA/400 μg NP**
*Cxcl1*	KC	Neutrophil chemokine	1.00+/-0.05	2.80+/-0.78**a**	2.48+/-0.28**b**	6.29+/-0.38**a,b**
*Cxcl2*	MIP-2	PMN chemokine	1.00+/-0.08	2.85+/-0.75**a**	4.22+/-0.76**b**	8.77+/-0.99**a,b**
*Cxcl10*	IP10	T-cell chemokine	1.00+/-0.05	1.17+/-0.05	14.32+/-3.95**b**	44.84+/-4.29**a,b**
*Ccl2*	MCP-1	Monocyte and T-cell chemokine	1.00+/-0.08	1.50+/-0.28	3.66+/-0.86**b**	13.84+/-1.71**a,b**
*Ccl8*	MCP-2	Monocyte chemokine	1.00+/-0.08	1.56+/-0.25	15.48+/-5.22**b**	105.12+/-32.47**a,b**
*Ccl11*	Eotaxin	Eosinophil chemokine	1.00+/-0.05	1.32+/-0.11	2.50+/-0.71	11.47+/-2.84**a,b**
*Itln1*	Itlna	Pathogen-associated molecular pattern recognition	1.00+/-0.25	4.95+/-1.78**a**	6.14+/-0.95**b**	38.65+/-13.92**a,b**
*Irg1*	Irg1	Toll like receptor response	1.00+/-0.14	4.70+/-1.15**a**	15.45+/-4.36**b**	154.18+/-16.97**a,b**
*Chi3l3*	YM1	Chitinase-like protein	1.00+/-0.07	1.01+/-0.12	1.30+/-0.26	1.94+/-0.28**b**
*Chi3l4*	YM2	Chitinase-like protein	1.00+/-0.35	4.61+/-2.98	9.13+/-3.96	33.10+/-12.56**b**
*Saa3*	Saa3	Acute phase protein	1.00+/-0.09	4.51+/-1.85	65.9+/-12.7**b**	157.88+/-19.38**a,b**
*Tnfα*	TNFα	Acute phase response cytokine	1.00+/-0.12	1.69+/-0.17**a**	2.20+/-0.23**b**	3.36+/-0.30**b**
*Il6*	IL6	Acute phase response cytokine	1.00+/-0.13	-1.14+/-0.13	2.00+/-0.40**b**	5.60+/-0.96**a,b**
*Il1β*	IL1β	Acute phase response cytokine	1.00+/-0.13	1.01+/-0.05	1.26+/-0.03	1.71+/-0.13**b**
*Il1rn*	IL1rn	IL1 receptor antagonist	1.00+/-0.04	1.13+/-0.07	1.70+/-0.23	4.62+/-0.71**a,b**
*Il2*	IL2	Th1 cytokine	1.00+/-0.06	1.06+/-0.07	1.57+/-0.24**b**	1.37+/-0.09
*Ifnγ*	IFNγ	Th1 cytokine	1.00+/-0.08	-1.06+/-0.14	1.67+/-0.28**b**	2.31+/-0.30**b**
*Il4*	IL4	Th2 cytokine	1.00+/-0.15	1.01+/-0.12	1.40+/-0.26	3.77+/-0.77**a,b**
*Il5*	IL5	Th2 cytokine	1.00+/-0.10	-1.20+/-0.18	2.39+/-0.59	7.17+/-1.44**a,b**
*Il13*	IL13	Th2 cytokine	1.00+/-0.10	1.34+/-0.39	6.80+/-2.96**b**	44.23+/-12.67**a,b**
*Retnla*	Fizz 1, HIMF	Th2 suppressor	1.00+/-0.12	1.32+/-0.37	6.88+/-1.78**b**	21.90+/-3.98**a,b**
*Il17a*	IL17A	Th17 cytokine	1.00+/-0.16	2.52+/-0.69	8.34+/-2.32**b**	46.24+/-6.02**a,b**
*Foxp3*	Foxp3	T-reg transcription factor	1.00+/-0.08	1.49+/-0.18	2.08+/-0.19**b**	3.98+/-0.33**a,b**
*Il10*	IL10	T-reg cytokine	1.00+/-0.09	1.09+/-0.17	2.97+/-0.76**b**	8.22+/-2.01**a,b**
*Muc5ac*	Muc5ac	Mucin production	1.00+/-0.19	1.61+/-0.61	4.72+/-1.44**b**	25.97+/-4.70**a,b**
*Clca3*	Gob5	Ion channel, mucus secretion	1.00+/-0.39	2.52+/-1.42	6.16+/-2.26	16.72+/-3.42**b**
*Clca2*	Clca2	Ion channel, fibrosis development	1.00+/-0.12	1.40+/-0.16	3.25+/-0.78**b**	7.96+/-1.20**a,b**
*Mmp12*	Mmp12	Macrophage elastase	1.00+/-0.08	3.06+/-0.81	1.83+/-0.22	7.91+/-0.59
*Timp1*	Timp1	Mmp inhibitor	1.00+/-0.05	-1.00+/-0.10	3.73+/-0.87**b**	12.40+/-1.68**a,b**

A qPCR analysis was performed on the genes that showed a ≥2 fold increase towards untreated controls in the gene array. **a**: Significant changes (p < 0.05) towards non-SNP exposed animals, **b**: significant changes towards non-OVA mice. The analysis reveals that neutrophil chemokines (*Kc, Cxcl1; Mip-2, Cxcl2*) and innate immune responsive genes (*Tnfα; Irg1; Itln1*) are significantly elevated by SNP exposure in both, SAL- and OVA-mice. A significant adjuvant SNP effect in SNP/OVA-mice was measured for Th2 cytokines (*Il4; Il5; Il3*) and Th17 associated genes (*Il17a; Il6*; *Saa3*). Monocyte and eosinophil chemokines (*Mcp-1*; *Mcp-2*; *Eotaxin*; *Ip10*) were further significantly increased in SNP/OVA-mice as well as other proteins which might be involved in tissue remodeling (*Fizz1*; *Timp1*). No adjuvant SNP effect was measured in Th1 cytokines (*Ifnγ*; *Il2*).

### Effects of SNP in SAL-mice

In animals that were exposed to SNP only (SNP-mice), there was a significant dose-dependent increase in neutrophils at 100 and 400 μg SNP with 3,210 ± 1,583 and 5,400 ± 1,999 neutrophils/mL BALF respectively, compared to non at 0 μg SNP (Figure 2B). No increases in BALF eosinophils and lymphocytes were detected (Figure 2C and D) and SNP exposure was not associated mucus airway remodeling (Figure 5E and F).

SNP exposure by itself, without OVA, resulted in significant gene expression of neutrophil chemokines *Kc* and *Mip-2*, as well as *Tnfα*, immunoresponsive gene 1 (*Irg1*) and intelectin1 (*Itln1*) (Table 2). In addition, there were significant increases of BALF chemokines KC and MIP-1α in SNP-mice (100 and 400 μg SNP) as compared to SAL-mice (Figure 8). These findings corresponded to the increase in BALF neutrophils in 100 and 400 μg SNP-mice (Figure 2B).

### Enhanced activation of tracheobronchial lymph node cells from SNP/OVA -mice

**Figure 9:**
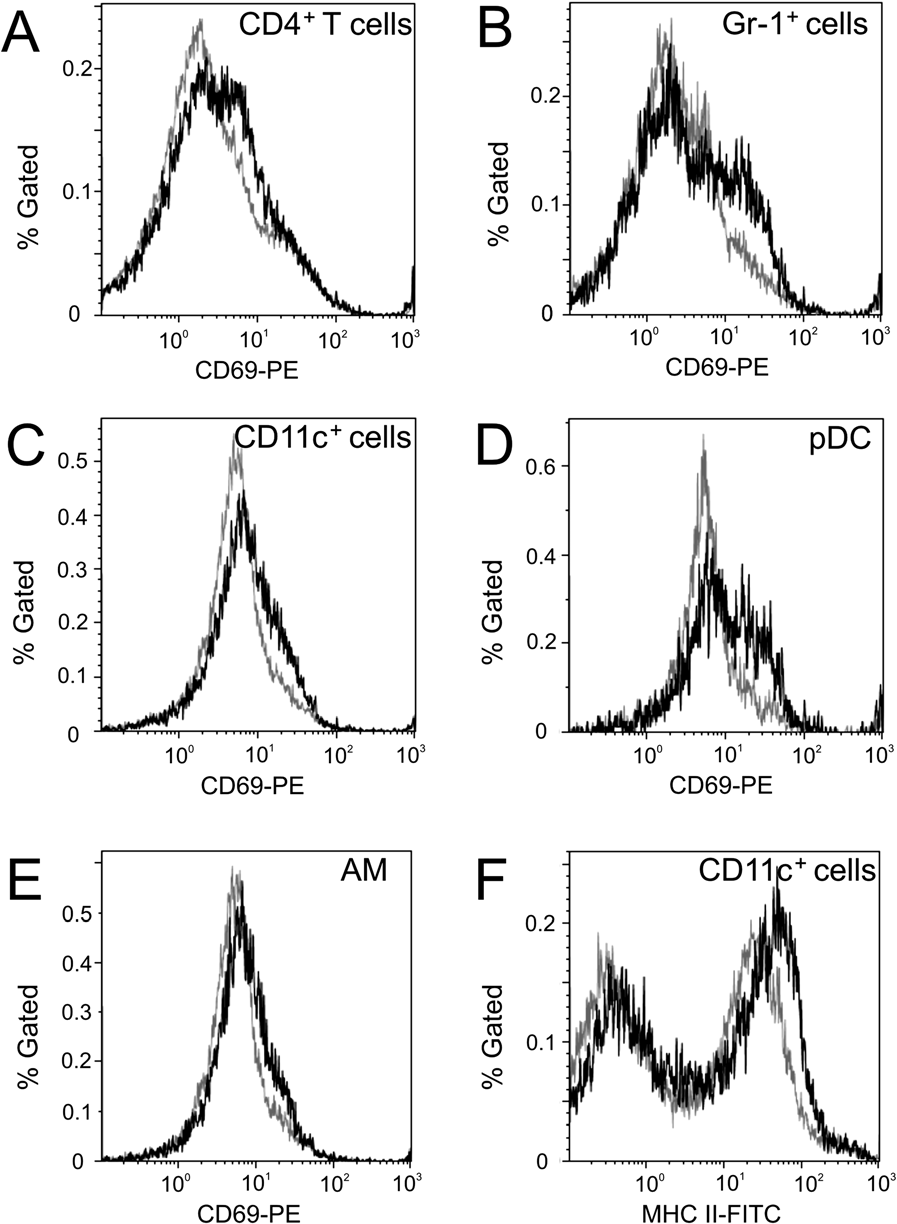
**Activation of immune cells from TBLN.** Cells from TBLN of OVA- and SNP/OVA-mice (10 μg SNP) were stained for expression of surface molecules, including CD4, CD69, CD11c, CD11b, MHC II, and Gr-1. Cells were gated on singlets using FSC-A/FSC-H, lymphocytes, monocytes, or granulocytes using FSC-A/SSC-A, and individual cell populations, including CD4^+^ cells, CD11c^+^, Gr-1^+^, CD11c^+^Gr-1^+^CD11b^-^ pDC, CD11c^+^Gr-1^-^CD11b^-^ AM. More details on the gating strategy are found in the supplementary information (Additional file 1: Figure S6). Expression of CD69 on CD4^+^ cells (**A**), Gr-1^+^ cells (**B**), CD11c^+^ cells (**C**), pDC (**D**), and AM (**E**), and expression of MHC II on CD11c^+^ cells (**F**) are presented in histograms, with X axis representing mean fluorescence intensity for CD69 or MHC II and Y for % of particular gated cell population. Six samples from OVA- (grey curve) or OVA/SNP- (black curve) mice were concatenated for flow cytometric analysis. Statistical analysis of percent values of CD69 expression from individual TBLN revealed a significant difference in CD11c^+^, AM, and pDC populations between OVA and SNP/OVA-mice.

To further investigate the effect of engineered SNP on lymphocytes and myeloid cells, activation of immune cell populations was assessed using tracheobronchial lymph node (TBLN) preparations. CD69 is constitutively expressed by platelets, mature thymocytes and monocytes, while it is induced on cells of hematopoietic lineages, including T and B lymphocytes, NK cells, murine macrophages, neutrophils, and eosinophils [19,20]. Therefore, CD69 was used as a marker to assess the activation status of cells in TBLN. The data within a group was concatenated and presented as a histogram (Figure 9). Statistically significant changes between OVA- and SNP/OVA-mice were tested with an unpaired *t*-test, comparing the percent CD69 expressing cells from individual animals. Apparent increases of CD69 expression on CD4^+^ T cells (Figure 9A) and Gr-1^+^ cells (Figure 9B) from SNP/OVA-mice (10 μg SNP) compared to OVA-mice, were not statistically significant. However, a significant (p > 0.05) increase for CD69 expression was observed on CD11c^+^ cells (Figure 9C). CD11c is expressed primarily on dendritic cells (DC) and macrophages, at lower levels on granulocytes, and least on T and B lymphocytes [21]. Gr-1 is a granulocyte marker, but is also expressed at moderate levels on plasmacytoid dendritic cells (pDC) in lymphoid tissues [22]. CD11c^+^ and Gr-1^+^ cell populations were further dissected to identify the cellular components that contributed to the increased expression of CD69 in SNP/OVA-mice. Interestingly, CD11c^+^Gr-1^+^CD11b^-^ pDC showed enhanced surface CD69 expression in the presence of SNP compared with the OVA alone group (Figure 9D). In addition, alveolar macrophages (AM) with the typical phenotype CD11c^+^Gr-1^-^CD11b^-^ exhibited the same CD69 expression profile (Figure 9E) [23]. The percent of CD69^+^ cells in both pDC and AM populations were statistically increased in the SNP/OVA-mice as compared with the OVA-mice (p < 0.05). Activation of lymphocytes was also studied by assessing maturation status of antigen presenting cells (APC), which was determined by expression levels of surface MHC II molecules [24]. In SNP/OVA-mice, CD11c^+^ cells also displayed a higher level of MHC II expression compared with the CD11c^+^ cells from OVA-mice (Figure 9F), though not statistically significant. In summary, immune cells from TBLN in SNP/OVA-mice exhibited a more elevated activation status compared with cells from OVA-mice.

## Discussion

The results of the present study indicate that airway exposure to engineered SNP during sensitization of mice to OVA enhances pathologic aspects of allergic airway disease upon secondary OVA challenge. This effect was more profound with increasing SNP doses and was characterized by enhancement of OVA-induced eosinophilic and lymphocytic inflammation, Th2 and Th17 cytokine expression, elevation of serum OVA-specific IgE and IgG1 levels, as well as an enhanced manifestation of mucous cell metaplasia in pulmonary airways. The fact that SNP exposure during allergen sensitization enhanced the pulmonary allergic response to the secondary OVA challenge indicates that SNP exhibits adjuvant-like characteristics in the development of allergic airway disease. Adjuvants are defined as compounds that are not immunogenic themselves, but increase and/or modulate the intrinsic immunogenicity of an antigen. Adjuvants are used in vaccines to induce potent and persistent immune responses, with the additional benefits that less antigen and/or fewer injections are needed [25]. In this study, however, SNP promoted the immunologic response towards the allergen (OVA) and thereby potentiated the adverse allergic responses in the pulmonary airways.

Our results suggest that workplace exposures to engineered NP could have similar adverse health consequences as those reported for UFP in outdoor air pollution associated with high traffic roadways. UFP collected from the Los Angeles Air Basin have been shown to act as adjuvants to enhance the development and severity of allergic airway disease, using a similar OVA-induced murine model of asthma [6]. In addition, epidemiologic studies have reported an increased incidence of asthma in children living in close proximity to highway traffic [26,27], where ambient UFP concentrations are high [28]. By comparison, relatively few studies have investigated the exacerbation of allergic airway disease by other types of NP exposures. Exacerbation in allergic airway disease, with increased Th2 responses, have been reported in OVA-sensitized and -challenged mice when co-exposed with 50 μg multi-walled carbon nanotubes (MWCNT) [29] or different sized carbon black NP [30]. Similar findings were reported by Hussain et al. in mice sensitized with toluene diisocyanate (TDI) via skin and exposed to TiO_2_ NP and gold NP via instillation (0.8 mg/kg body weight) [31]. Unlike our study design, mice in these studies were co-exposed to NPs during both the sensitization and challenge phases of the allergen administration. In our study we found that inhaled SNP during sensitization phase alone can act as adjuvants to markedly increase the magnitude of the host’s secondary immune response upon subsequent allergen challenge. A similar effect was also found previously for nano-sized crystalline silica particles [32]. However, a recent study by Ban and colleagues [33] addressed the same question in a similar OVA mouse model with iron oxide NP, and found that iron NP exposure during sensitization with OVA results in attenuation of OVA mediated allergic airway disease. This suggests that the adjuvant effect of SNP in the development of allergic airway disease, we observed in our study, is likely to be particle specific. Further research, however, is needed to understand the immune modulatory effects of different NP and the impact of NP material, size and surface coating.

In our study, SNP exposure alone, without the antigen, caused a dose-dependent pro-inflammatory response in non-allergic animals as indicated by a modest increase in BALF neutrophils and elevations of neutrophil-related chemokines and innate immune response genes, namely *Kc*, *Mip-1α*, *Mip-2*, *Itln1* and *Irg1*. These effects were evident at SNP exposure doses of 100 and 400 μg and demonstrate that these engineered SNP at high dose cause a minimal, yet sustained, innate immune responses up to 16 days post-instillation. By comparison, many rodent SNP toxicity studies describe airway neutrophilic inflammation that is accompanied by overt toxicity and tissue injury. For example, persistent pulmonary toxicity [13,34], including neutrophilic inflammation, apoptosis, tissue injury [13,35], the induction of pro-inflammatory BALF cytokines such as IL1β, IL6 and TNFα [34] as well as cardiovascular effects [36] have been reported. These studies used smaller sized SNP (14 nm) at similar or higher exposure doses (100 μg/mouse [13]; 3 mg/mouse [35]). Compared to these *in vivo* studies, we used larger SNP (90 nm), which appear to be less toxic than smaller SNP reported by others [9]. The SNP used in ours study were further modified with a PEG shell which prevents them from agglomeration and gives them the ability to penetrate rapidly through airway mucus barriers [37]. PEG-coatings have been reported to decrease systemic NP interactions and overall toxicity [38-40], which might explain the less severe acute inflammatory response we observed compared to those reported by others.

Adjuvant effects described in the current study had a mixed Th2/Th17 cytokine response, similar to findings by Li et al. who used ambient UFP in a similar OVA model [6]. Mechanisms by which SNP may induce an adjuvant Th2/Th17 cytokine response can only be speculated, since this study was not designed to investigate the underlying mechanisms of adjuvancy in detail. SNP are known to induce oxidative stress [9], which plays an important role in the pathogenesis of asthma [7,41]. This connection has been shown by Li et al. for ambient UFP where co-administration with the anti-oxidant N-acetylcysteine in OVA challenged mice diminished the adjuvant allergic airway response of UFP [7]. In another study, oxidative stress, caused by diesel enriched PM, was also shown to skew the immune response from a Th1 to a Th2 cytokine profile [42]. It has been suggested that oxidative stress-induced activation of transcription factor NF-E2-related factor 2 (NRF2), during the sensitization phase with an allergen, can down regulate the production of Th1 cytokines IL12 and IFNγ [43] and thereby leads to a Th1/Th2 imbalance. Furthermore results from *in vitro* studies on amorphous, colloidal SNP in a size range of 14 to 80 nm show a size- and dose-dependent cytotoxicity of SNP with induction of oxidative stress and/or glutathione (GSH) depletion [44,45]. At the time point after challenge, however, we did not detect any signs of oxidative stress. Further investigations are therefore needed to assess oxidative stress and Th1/Th2 cytokine balance during the sensitization phase of our protocol in order to address these potential mechanisms of SNP-associated adjuvancy.

Recently IL17 has been associated with more severe forms of asthma, especially those cases complicated by persistent airway neutrophils [46]. Th17 responses in allergic airway disease are promoted by IL6, tumor growth factor β (TGFβ), IL23, SAA3 as well as IL1β [47,48]. In our study we detected significant increases in allergic SNP/OVA-mice of IL17A, IL6 and IL1β (cytokine and gene expression) as well as *Saa3* (gene expression). SAA3 has been shown to activate the NLRP3 inflammasome and promote an allergic Th17 response in mice in combination with other mediators [47]. The NLRP3 inflammasome is a protein complex required for splicing pro-IL1β into its active form [49], and was recently found to have an immuno-stimulatory function for aluminum adjuvants in vaccination [50]. Though we did not measure inflammasome activation in the present study, SNP treatment caused a dose- dependent increase in BALF IL1β, and a recent study has shown that SNP can activate the inflammasome [51]. It is therefore possible that the SNP-mediated adjuvant effects in allergic models are related to inflammasome activation and/or oxidative stress. However, further studies are needed to elucidate their roles in the adjuvant effects of SNP in more detail.

Besides Th2 and Th17 cytokine responses, we also detected an increase in TNFα and Th1 cytokine IFNγ in the SNP/OVA-mice which was not detectable in SNP- or OVA-mice. Therefore it may be possible that a Th1 component is present as well in the adjuvant response of SNP/OVA-mice. It has been shown previously, that Th1 cells do not attenuate Th2 cell–induced airway hyperreactivity in OVA-immunized BALB/c mice, but rather cause severe airway inflammation [52]. We therefore suggest that the increase in BALF cytokines TNFα and IFNγ are augmenting rather than attenuating the allergic response in our model.

Besides stimulation of Th2 response, NP have also been shown to influence maturation, antigen presentation and co-stimulation of DC [53], and the analysis of TBLN cell populations in our study confirmed these findings. In response to DNA and RNA viruses, pDC secrete large amounts of IFN-α and IFN-β that play important roles in activating other cells in the immune system. For example, IFN-α and IFN-β produced by pDC have been shown to increase CD69 expression and IFN-γ production from CD4^+^ T cells [54,55], and also activate CD8^+^ T cells upon influenza challenge [56]. Up-regulation of CD69 on pDC in response to influenza infection has been found to cause down-regulation of sphingosine-1-phosphate (S1P) on pDC, resulting in elongated transit time of pDC and their accumulation in LN [57]. An OVA-induced CD69 up-regulation on pDC might therefore lead to retention of pDC in TBLN where they could interact with lymphocytes and stimulate an immune response. The CD69 expression on pDC was further increased by co-exposure with SNP. Although AM have been suggested to prevent development of airway hyperresponsiveness upon OVA challenge [58], AM are also known to produce proinflammatory cytokines that enhance Th2 cytokine production by pulmonary CD4^+^ T lymphocytes [59]. In our study, the activation of AM was further increased in the presence of SNP and an increase in BALF Th2 cytokines was measured. In addition, OVA-induced maturation of CD11c^+^ cells, including APC, such as DC and macrophages, was also exaggerated by SNP. Koike et al. further confirmed that effects on APC parallel those on allergic pathology *in vivo* in their overall trend [60]. These mechanisms might partly explain the SNP-mediated immune enhancement; however, further research is still required to understand interaction of NPs with the immune system, which could include additional surface markers to further refine effects of NPs on various myeloid subpopulations.

In our treatment protocol, mice were IN instilled with a wide range of SNP doses (0, 10, 100 or 400 μg SNP per instillation). The applied doses of SNP used in our study are comparable to those used in similar studies performing intratracheal (IT) or IN administration [13,29,30,32,33]. IN has been shown to be an easily applicable and efficient method to administer particle suspensions in murine models [61]. Nevertheless, particle inhalation represents a more realistic model for NP workplace exposure. Intrapulmonary NP delivery and distribution might differ between inhalation and instillation exposure which may result in different dose responses. The potential workplace exposure levels for SNP, however, are currently not known and it is therefore difficult to estimate a realistic dosing regimen. It has been reported that airborne, crystalline silica particles may reach as high as 0.28 mg/m^3^ in some workplace conditions such as cement mason/concrete finisher [62].

In summary, the results of our study indicate that engineered SNP can act as adjuvants to enhance the development of allergic airway disease in mice. This finding further suggests that individuals exposed to SNP might be more prone to develop allergic airway diseases, establishing a new aspect of NP toxicity that has particular relevance to occupational NP exposure. More research, however, is needed to clarify the potential risks of NP exposure in the development of allergic airway diseases in humans. Nevertheless, the murine allergic OVA model we used in our study, which involved IN instillation of allergen and SNPs during sensitization followed by IN challenge with allergen only, may be used to test the adjuvant potential of other NPs in allergic airway disease.

## Material and methods

### Particle preparation and characterization

Plain SNP (LUDOX^®^ TM-40 colloidal silica, 40 wt% suspension in H_2_O), aminopropyltriethoxysilane (APTES), propargyl chloroformate and sodium azide were purchased from Sigma-Aldrich. Anhydrous dimethylformamide (DMF) was freshly distilled from powered BaO. Cu(PPh_3_)Br and 1-azido-2-(2-(2-(2-methoxyethoxy)ethoxy)ethoxyethane (PEG-N_3_) were prepared as previously described in detail [63,64]. PEG-modified SNP were synthesized from commercially available LTM40 SNP in three steps (shown in Additional file 1: Figure S1). To avoid aggregation, the modified SNP were purified by several steps of washing the NP with solvent, followed by centrifugation. Resulting particles were used directly to the following step and aggressive drying such as drying under vacuum was avoided [65]. First, APTES was condensed on plain SNP. The resulting amine-modified SNP (aSNP) were washed twice with reagent grade DMF, and then three times with anhydrous DMF to remove ethanol and water. aSNP were reacted with propargyl chloroformate to afford the alkyne-modified particles (aaSNP) which were purified by centrifugation. The resulted particles were clicked with PEG-N_3_ catalyzed by 10% of Cu(PPh_3_)Br in DMF. The resulted SNP were purified by centrifugation to receive the PEG-coated SNP. Note that the SNP suspension was not tested for endotoxin content. It has been shown previously that LPS exposure during sensitization with OVA might suppress the development of a Th2 cytokine response [66], which was not the case in our study.

Dynamic Light Scattering (DLS) was performed with a Malvern NanoZS ZetaSizer to measure the particle hydrodynamic size and intensity average diameters. The particle samples for DLS analyses were sonicated prior to DLS measuring. Particle size, surface grafting amount at different step of synthesis are shown in Table 1. The final SNP used for the animal exposure study had a hydrodynamic diameter of 90 nm. Zeta potential of the particles was not measured, however, the size distribution measurements of the particles (Additional file 1: Figure S4) only shows a single peak at 90 nm and therefore suggesting single SNP dispersion. A more detailed description of particle synthesis and characterization can be found in the supplementary information (Additional file 1).

### Experimental protocol

Female BALB/c mice (~20 grams body weight; 6–8 weeks old) were obtained from Charles River (Portage, MI). Mice were maintained at the Michigan State University (MSU) animal housing facilities at room temperature of 21°C–24°C and relative humidity of 45–70%, with a 12 h light/dark cycle starting at 7:30 A.M. All animal procedures and experimental protocols were approved by the MSU Institutional Animal Care and Use Committee; MSU is an AAALAC accredited institution.

On days 1, 3, 6 and 8, BALB/c mice were intranasally sensitized with 0.02% OVA (allergen; Sigma-Aldrich) in saline or saline alone (vehicle control) at a volume of 15 μL per nostril (total intranasal volume of 30 μL) (n = 6 animals/group). SNP were co-administered with intranasal doses of 0, 10, 100, or 400 μg (Figure 1). On days 22 and 23, OVA-sensitized mice were challenged intranasally with 30 μL of a 0.5% OVA in saline solution. All mice were anesthetized with 4% isoflurane prior to each intranasal aspiration. Animals were sacrificed 24 hours after the last intranasal challenge (Day 24).

### Necropsy, lavage collection and tissue preparation

Mice were anesthetized with an intraperitoneal injection of sodium pentobarbital (60 mg/kg body weight). A midline laparotomy was performed and approximately 0.5 mL of blood was drawn from the vena cava and collected in heparinized tubes (BD Microtainer, Franklin Lakes, NJ) for separation of plasma. Animals were exsanguinated via the abdominal aorta. Immediately after death, the trachea was exposed and cannulated and the heart and lungs were excised en bloc. A volume of 0.8 mL sterile saline was instilled through the tracheal cannula and withdrawn to recover bronchoalveolar lavage fluid (BALF). A second intratracheal saline lavage was performed and the collected BALF was combined with the first sample for analysis.

After the BALF was collected, the right lung lobes were ligated, removed and placed in RNA*later* (Qiagen, CA). Samples were kept at - 20°C until further processing for RNA isolation. The left lung lobe was intratracheally fixed with neutral-buffered formalin at a constant pressure (30 cm H_2_O) for 2 h and then stored in a large volume of the same fixative until further tissue processing for light microscopy.

Twenty-four hours later, two sections were excised at the level of the 5th and 11th airway generation along the main axial airway (G5 and G11), to sample proximal and distal bronchiolar airways, respectively [67]. Tissue blocks were then embedded in paraffin and 5- to 6-μm-thick sections were cut from the anterior surface. Lung sections were stained with hematoxylin and eosin (H&E) for routine light microscopic examination and with Alcian Blue (pH 2.5)/Periodic Acid–Schiff (AB/PAS) for identification of intraepithelial neutral and acidic mucosubstances in pulmonary bronchiolar epithelium. To detect eosinophils, slides were immunostained using a polyclonal rabbit antibody directed against murine eosinophil-specific major basic protein (MBP; 1:500; Mayo Clinic, AZ).

In OVA- and OVA/SNP-mice (10 μg SNP), TBLN were removed *en bloc* from the mice, and homogenized in 1× PBS. Single-cell suspensions were prepared, and 1 × 10^6^ cells from each sample were collected in a 96 well round-bottom plate for flow cytometric analysis.

### BALF cytometry

Total number of cells in the collected BALF was estimated using a hemocytometer. All intact cells were counted, but no trypan blue exclusion test of dead cells was performed. Cytological slides were prepared by centrifugation at 400 g for 10 min using a Shandon cytospin 3 (Shandon Scientific, PA) and stained with Diff-Quick (Dade Behring, DE). Differential cell counts for neutrophils, eosinophils, macrophages/monocytes, and lymphocytes were assessed from a total of 200 cells. Remaining BALF was centrifuged at 2400 g for 15 min and the supernatant fraction was collected and stored at -80°C for later biochemical analysis.

### Flow cytometric analyses for inflammatory cytokines

BALF supernatants were assayed for the inflammatory cytokines IL-1β, IL-2, IL4, IL-5, IL-6, IL-13, IL-17A, TNFα, IFN-γ, MCP-1, MIP-1α and KC. All cytokine kits were purchased as either Flex Set reagents or as preconfigured cytometric bead array kits (BD Biosciences, San Jose, CA). Cytokine analysis was performed using a FACSCalibur flow cytometer (BD Biosciences). 50 μL of BALF was added to the antibody-coated bead complexes and incubation buffer. Phycoerythrin-conjugated secondary antibodies were added to form sandwich complexes. After acquisition of sample data using the flow cytometer, cytokine concentrations were calculated based on standard curve data using FCAP Array software (BD Biosciences).

### Flow cytometric analyses for surface markers

Cells from TBLN were incubated with purified rat anti-mouse CD16/CD32 (Fc block; BD Pharmingen, San Diego, CA) in FACS buffer (1× HBSS, 1% bovine serum albumin (BSA), 0.1% sodium azide, pH 7.6). Cells were then stained for surface markers using following antibodies from Biolegend (San Diego, CA): phycoerythrin (PE)/cy7-conjugated anti-mouse CD4 (clone RM4-5), Pacific Blue-conjugated anti-mouse CD11b (clone M1/70), allophyeocyanin (APC)/cy7-conjugated anti-mouse CD11c (clone N418), fluorescein isothiocyanate (FITC)-conjugated anti-mouse MHC II (I-A/I-E, clone M5/114.15.2), APC-conjugated anti mouse Gr-1 (granulocyte differentiation antigen 1, clone RB6-8C5), and PE-conjugated anti-mouse CD69 (clone H1.2 F3). Cells were fixed with Cytofix (BD Pharmingen) and analyzed using a FACSCanto II flow cytometer (BD Biosciences, San Jose, CA). A graph, explaining the applied gating strategy, is presented in the supplementary information (Additional file 1: Figure S6). Samples were concatenated (n = 6) and further analyzed using Kaluza 1.1 software (Beckman Coulter, Miami, FL). The fluorescence intensity values for CD69 or MHC II expression by different populations were presented in histograms. To determine the statistically significant changes between OVA and SNP/OVA groups, an unpaired *t*-test was performed on non-concatenated data. The percent values of CD69 expression by CD4^+^, CD11c^+^, Gr-1^+^, AM, and pDC populations, respectively, or the percent values of MHC II expression by CD11c^+^ population from individual animal were transformed and analyzed using GraphPad Prism v4.0 (Graphpad Software, San Diego, CA).

### ELISA assay OVA- IgE/IgG1

OVA-specific IgE and IgG1 expression in serum was analyzed with an ELISA kit (BD PharMingen, CA) as described previously [6]. 96 well plates (Costar, NY) were coated overnight with 50 μg/mL OVA. After washing with phosphate buffered saline (PBS) and blocking with 10% fetal bovine serum (FBS)/PBS, samples and standards were loaded and incubated overnight. Following additional washing steps, detection antibody (biotin-conjugated rat anti-mouse IgG1 or IgE Antibody (BD PharMingen, CA) was applied and incubated for 4 h. Finally, streptavidin-horseradish peroxidase mixture was added for 30 min, followed by 20 min incubation with reaction substrate. Optical density (OD) was read at 405 nm wave length with a Spectra Max Gemini plate reader (Molecular Devices, CA). The OVA-IgG1 standard was a monoclonal anti-chicken egg albumin (Sigma-Aldrich, MO). Since no standard was available for IgE, OVA-specific IgE was determined only by OD. All samples were loaded and measured on the same 96-well plate, therefore a standard was not essential for comparing relative increases in IgE concentrations.

### Real-time PCR of pulmonary tissues

Total RNA was isolated from right lung lobes using RNeasy Mini Kit (Qiagen, CA) according to the manufacturer’s instructions. Briefly, tissues were homogenized in lysis buffer (Buffer RLT) containing 2-mercaptoethanol with a 5 mm rotor-stator homogenizer (PRO Scientific, CT). During RNA purification, DNase digestion was performed on-column using Qiagen RNase-Free DNase Set. Purified RNA was quantified using a GeneQuant Pro spectrophotometer (BioCrom, England). CDNA was generated from 2 μg of total RNA using the High-Capacity cDNA Reverse Transcription Kit. The reaction mixture was incubated at 25°C for 10 min and then 37°C for 2 h. PCR array analysis was performed by pooling aliquots of cDNAs from samples in each experimental group. Quantitative gene expression analysis was performed using *Taq*Man Gene Expression Assay reagents on the ABI PRISM 7900 HT Sequence Detection System. The PCR cycling parameters were 48°C for 2 min, 95°C for 10 min, and 50 cycles of 95°C for 15 s followed by 60°C for 1 min. Relative gene expression levels were reported as fold-change using the ΔΔC_t_ method where FC = 2^-ΔΔCt^. The mRNA expression of each gene was normalized by subtracting the geometric mean of the C_t_s from four endogenous controls (*Actb, Arbp, Gapd, Gusb*). Selected genes that had expression levels at least 2-fold greater in experimental groups relative to the control group were confirmed by relative quantitative real-time RT-PCR using individual animal cDNAs as described above. Statistical differences between ΔC_t_ values of different groups were determined with two-way ANOVA (SigmaStat, Ashburn, VA; *P* ≤ 0.05).

### Airway morphometry

Morphometric estimation of the amount of intraepithelial AB/PAS mucosubstances was conducted as previously described [68]. These quantitative analyses were performed using Scion Image (Scion Corporation, MD), to estimate the volume density (VD_muc_) of AB/PAS stained mucosubstances stored in mucus-secreting cells of the bronchiolar epithelium lining the axial airways G5 and G11 and using the equation below. The area of mucosubstance (A_muc_) in the respiratory epithelium lining the cross-sections of selected axial airways was sampled within a random field of interest and correlated to the corresponding length of basal lamina (L_BL_) (Equation 1.)

(1) $${\mathrm{VD}}_{\text{muc}}\;=\;\frac{A_{\text{muc}}}{L_{\mathrm{BL}}\times 4/\prod }$$

### Statistics

Each study group consisted of 6 mice and all data were reported as group means ± standard error of the mean (SEM). Grubbs outlier test was performed and recognized outliers were removed from the analysis. Differences among groups were analyzed by a two-way ANOVA followed by a pair-wise comparison (Student-Newman-Keuls). When normality or variance equality failed, a Kruskal-Wallis ANOVA on ranks was performed. All analyses were conducted using SigmaStat software (SigmaStat; Jandel Scientific, San Rafael, CA). Significance was assigned to *p* values less than or equal to 0.05.

## Abbreviations

aaSNP: Alkyne-modified silica nanoparticles; aSNP: Amine-modified silica nanoparticles; AB/PAS: Alcian Blue/Periodic Acid–Schiff; Actb: β-actin; AM: Alveolar macrophages; Amuc: Area of mucus; APC: Antigen presenting cell; APTES: Aminopropyltriethoxysilane; Arbp: Acidic ribosomal phosphoprotein P0; BALF: Bronchoalveolar lavage fluid; CCL: Chemokine (C-C motif) ligand; CXCL: Chemokine (C-X-C motif) ligand; DC: Dendritic cells; DMF: Dimethylformamide; FBS: Fetal bovine serum; FoxP3: Forkhead protein P3; G5/G11: Histological sections at the 5th and 11th airway generation of the main axial airway; Gapdh: Glyceraldehyde-3-phosphate dehydrogenase; Clca3: Chloride channel calcium activated 3 (Gob5); GSH: Glutathione; Gusb: β-glucuronidase; IgE: Immunoglobulin isotype E; IgG1: Immunoglobulin isotype G1; IL: Interleukin; IFNγ: Interferon γ; IP10: Inflammatory protein (CXCL10); KC: (CXCL1) keratinocyte chemoattractant; LBL: Length of basal lamina; LTM40: LUDOX^®^ TM-40 colloidal silica 40 wt% suspension in H_2_O; MBP: Major basic protein; MCP-1: (CCL2) monocyte chemoattractant protein 1; MCP-2: (CCL8) monocyte chemoattractant protein 2; MIP-1α: (CXCL3) macrophage inflammatory protein 1 alpha; MMP12: Matrix metalloproteinase 12; MUC5AC: Mucin 5 AC; OD: Optical density; OVA: Ovalbumin; PBS: Phosphate buffered saline; PEG: Polyethylene glycol; PM: Particulate matter; Saa3: Serum amyloid A3; SNP: Silica nanoparticles; TBLN: Tracheobronchial lymph nodes; TIMP1: Tissue inhibitor of metalloproteinase 1; TNFα: Tumor necrosis factor α; T-reg: Regulatory T cells; UFP: Ultrafine particles; VDmuc: Volume density of mucus.

## Competing interests

The authors declare that they have no competing interests.

## Authors’ contributions

CB analyzed and interpreted the data and wrote major parts of the manuscript; NLR, DNJH and LAB performed animal exposure, animal necropsy and BALF cell and cytokine experiments; NLR performed airway morphometry; QZ and GLB designed and characterized the particles; CB and RPL conducted gene expression analysis; JGW did IgG1 and IgE ELISAs; WC and BLK performed tracheobronchial lymph nodes experiments; NEK and RMW made substantial contributions to the analysis and interpretation of the data; JRH conceived the study design, performed histopathology and wrote parts of the manuscript. All of the authors critically read and approved the final manuscript.

## Supplementary Material

Additional file 1 **Additional information on material and methods.** Further information on the design of engineered silica nanoparticles and on the gene expression analysis of the lung tissue are provided in the Additional file 1, as well as more detailed information on the gating strategy for FACS. Click here for file

## References

[B1] Maynard AD Nanotechnology: the next big thing, or much ado about nothing? Ann Occup Hyg 2007 51 1 12 17041243 10.1093/annhyg/mel071

[B2] Kuhlbusch TA Asbach C Fissan H Göhler D Stintz M Nanoparticle exposure at nanotechnology workplaces: a review Part Fibre Toxicol 2011 8 22 10.1186/1743-8977-8-22 21794132 PMC3162892

[B3] Eisen EA Costello S Chevrier J Picciotto S Epidemiologic challenges for studies of occupational exposure to engineered nanoparticles; a commentary J Occup Environ Med 2011 53 S57 S61 21654419 10.1097/JOM.0b013e31821bde98

[B4] Inoue K-I Takano H Aggravating impact of nanoparticles on immune-mediated pulmonary inflammation ScientificWorldJournal 2011 11 382 390 21336454 10.1100/tsw.2011.44 PMC5596528

[B5] Peden D Reed CE Environmental and occupational allergies J Allergy Clin Immunol 2010 125 S150 S160 10.1016/j.jaci.2009.10.073 20176257

[B6] Li N Harkema JR Lewandowski RP Wang M Bramble LA Gookin GR Ning Z Kleinman MT Sioutas C Nel AE Ambient ultrafine particles provide a strong adjuvant effect in the secondary immune response: implication for traffic-related asthma flares Am J Physiol Lung Cell Mol Physiol 2010 299 L374 L383 10.1152/ajplung.00115.2010 20562226 PMC2951067

[B7] Li N Wang M Bramble LA Schmitz DA Schauer JJ Sioutas C Harkema JR Nel AE The adjuvant effect of ambient particulate matter is closely reflected by the particulate oxidant potential Environ Health Perspect 2009 117 1116 1123 19654922 10.1289/ehp.0800319 PMC2717139

[B8] Rückerl R Schneider A Breitner S Cyrys J Peters A Health effects of particulate air pollution: a review of epidemiological evidence Inhal Toxicol 2011 23 555 592 10.3109/08958378.2011.593587 21864219

[B9] Napierska D Thomassen LCJ Lison D Martens JA Hoet PH The nanosilica hazard: another variable entity Part Fibre Toxicol 2010 7 39 10.1186/1743-8977-7-39 21126379 PMC3014868

[B10] Ding M Chen F Shi X Yucesoy B Mossman B Vallyathan V Diseases caused by silica: mechanisms of injury and disease development Int Immunopharmacol 2002 2 173 182 10.1016/S1567-5769(01)00170-9 11811922

[B11] Arts JHE Schijf MA Kuper CF Preexposure to amorphous silica particles attenuates but also enhances allergic reactions in trimellitic anhydride-sensitized brown norway rats Inhal Toxicol 2008 20 935 948 10.1080/08958370802105371 18668410

[B12] Johnston CJ Driscoll KE Finkelstein JN Baggs R O’Reilly MA Carter J Gelein R Oberdörster G Pulmonary chemokine and mutagenic responses in rats after subchronic inhalation of amorphous and crystalline silica Toxicol Sci 2000 56 405 413 10.1093/toxsci/56.2.405 10911000

[B13] Kaewamatawong T Shimada A Okajima M Inoue H Morita T Inoue K Takano H Acute and subacute pulmonary toxicity of low dose of ultrafine colloidal silica particles in mice after intratracheal instillation Toxicol Pathol 2006 34 958 965 10.1080/01926230601094552 17178696

[B14] Lee KP Kelly DP The pulmonary response and clearance of Ludox colloidal silica after a 4-week inhalation exposure in rats Fundam Appl Toxicol 1992 19 399 410 10.1016/0272-0590(92)90179-L 1334015

[B15] Ryan SM Mantovani G Wang X Haddleton DM Brayden DJ Advances in PEGylation of important biotech molecules: delivery aspects Expert Opin Drug Deliv 2008 5 371 383 10.1517/17425247.5.4.371 18426380

[B16] Nakanishi A Morita S Iwashita H Sagiya Y Ashida Y Shirafuji H Fujisawa Y Nishimura O Fujino M Role of gob-5 in mucus overproduction and airway hyperresponsiveness in asthma Proc Natl Acad Sci USA 2001 98 5175 5180 10.1073/pnas.081510898 11296262 PMC33183

[B17] Vermeer PD Denker J Estin M Moninger TO Keshavjee S Karp P Kline JN Zabner J MMP9 modulates tight junction integrity and cell viability in human airway epithelia Am J Physiol Lung Cell Mol Physiol 2009 296 L751 L762 10.1152/ajplung.90578.2008 19270179 PMC2681350

[B18] Calvo FQ Fillet M De Seny D Meuwis M-A Maree R Crahay C Paulissen G Rocks N Gueders M Wehenkel L Merville M-P Louis R Foidart J-M Noël A Cataldo D Biomarker discovery in asthma-related inflammation and remodeling Proteomics 2009 9 2163 2170 10.1002/pmic.200800643 19322781

[B19] Marzio R CD69 And regulatiof the immune function Immunopharmacol Immunotoxicol 1999 21 565 582 10.3109/08923979909007126 10466080

[B20] Ziegler S Ramsdell F Alderson M The activation antigen CD69 Stem Cells 1994 12 456 465 10.1002/stem.5530120502 7804122

[B21] Sadhu C Ting HJ Lipsky B Hensley K Garcia-Martinez LF Simon SI Staunton DE CD11c/CD18: novel ligands and a role in delayed-type hypersensitivity J Leukoc Biol 2007 81 1395 1403 10.1189/jlb.1106680 17389580

[B22] Nakano H Yanagita M Gunn MD CD11c + B220+ Gr-1+ cells in mouse lymph nodes and spleen display characteristics of plasmacytoid dendritic cells J Exp Med 2001 194 1171 1178 10.1084/jem.194.8.1171 11602645 PMC2193516

[B23] Voisin M-B Buzoni-Gatel D Bout D Velge-Roussel F Both expansion of regulatory GR1 + CD11b + myeloid cells and anergy of T lymphocytes participate in hyporesponsiveness of the lung-associated immune system during acute toxoplasmosis Infect Immun 2004 72 5487 5492 10.1128/IAI.72.9.5487-5492.2004 15322051 PMC517443

[B24] Al-Daccak R Mooney N Charron D MHC class II signaling in antigen-presenting cells Current Opin Immunol 2004 16 108 113 10.1016/j.coi.2003.11.006 14734118

[B25] Guy B The perfect mix: recent progress in adjuvant research Nat Rev Microbiol 2007 5 505 517 10.1038/nrmicro1681 17558426

[B26] Chang J Delfino RJ Gillen D Tjoa T Nickerson B Cooper D Repeated respiratory hospital encounters among children with asthma and residential proximity to traffic Occup Environ Med 2009 66 90 98 19151227 10.1136/oem.2008.039412

[B27] Price K Plante C Goudreau S Boldo EIP Perron S Smargiassi A Risk of childhood asthma prevalence attributable to residential proximity to major roads in Montreal, Canada Can J Public Health 2012 103 113 118 22530532 10.1007/BF03404213 PMC6974016

[B28] Sioutas C Delfino RJ Singh M Exposure assessment for atmospheric ultrafine particles (UFPs) and implications in epidemiologic research Environ Health Perspect 2005 113 947 955 10.1289/ehp.7939 16079062 PMC1280332

[B29] Inoue K-I Koike E Yanagisawa R Hirano S Nishikawa M Takano H Effects of multi-walled carbon nanotubes on a murine allergic airway inflammation model Toxicol Appl Pharmacol 2009 237 306 316 10.1016/j.taap.2009.04.003 19371758

[B30] Inoue K-I Takano H Yanagisawa R Ichinose T Sakurai M Yoshikawa T Effects of nano particles on cytokine expression in murine lung in the absence or presence of allergen Arch Toxicol 2006 80 614 619 10.1007/s00204-006-0075-3 16482471

[B31] Hussain S Vanoirbeek JAJ Luyts K De Vooght V Verbeken E Thomassen LCJ Martens JA Dinsdale D Boland S Marano F Nemery B Hoet PHM Lung exposure to nanoparticles modulates an asthmatic response in a mouse model Eur Respir J 2011 37 299 309 10.1183/09031936.00168509 20530043

[B32] Han B Guo J Abrahaley T Qin L Wang L Zheng Y Li B Liu D Yao H Yang J Li C Xi Z Yang X Adverse effect of nano-silicon dioxide on lung function of rats with or without ovalbumin immunization PLoS One 2011 6 e17236 10.1371/journal.pone.0017236 21359146 PMC3040772

[B33] Ban M Langonné I Huguet N Guichard Y Goutet M Iron oxide particles modulate the ovalbumin-induced Th2 immune response in mice Toxicol Lett 2013 216 31 39 10.1016/j.toxlet.2012.11.003 23147377

[B34] Chen Z Meng H Xing G Yuan H Zhao F Liu R Chang X Gao X Wang T Jia G Ye C Chai Z Zhao Y Age-related differences in pulmonary and cardiovascular responses to SiO2 nanoparticle inhalation: nanotoxicity has susceptible population Environ Sci Technol 2008 42 8985 8992 10.1021/es800975u 19192829

[B35] Cho W-S Choi M Han BS Cho M Oh J Park K Kim SJ Kim SH Jeong J Inflammatory mediators induced by intratracheal instillation of ultrafine amorphous silica particles Toxicol Lett 2007 175 24 33 10.1016/j.toxlet.2007.09.008 17981407

[B36] Brown SC Kamal M Nasreen N Baumuratov A Sharma P Antony VB Moudgil BM Influence of shape, adhesion and simulated lung mechanics on amorphous silica nanoparticle toxicity Advanced Powder Technol 2007 18 69 79 10.1163/156855207779768214

[B37] Lai SK O’Hanlon DE Harrold S Man ST Wang Y-Y Cone R Hanes J Rapid transport of large polymeric nanoparticles in fresh undiluted human mucus Proc Natl Acad Sci USA 2007 104 1482 1487 10.1073/pnas.0608611104 17244708 PMC1785284

[B38] Carvalho LV Ruiz RDC Scaramuzzi K Marengo EB Matos JR Tambourgi DV Fantini MCA Sant’Anna OA Immunological parameters related to the adjuvant effect of the ordered mesoporous silica SBA-15 Vaccine 2010 28 7829 7836 10.1016/j.vaccine.2010.09.087 20937318

[B39] Díaz B Sánchez-Espinel C Arruebo M Faro J De Miguel E Magadán S Yagüe C Fernández-Pacheco R Ibarra MR Santamaría J González-Fernández A Assessing methods for blood cell cytotoxic responses to inorganic nanoparticles and nanoparticle aggregates Small 2008 4 2025 2034 10.1002/smll.200800199 18855973

[B40] Brandenberger C Mühlfeld C Ali Z Lenz A-G Schmid O Parak WJ Gehr P Rothen-Rutishauser B Quantitative evaluation of cellular uptake and trafficking of plain and polyethylene glycol-coated gold nanoparticles Small 2010 6 1669 1678 10.1002/smll.201000528 20602428

[B41] Holguin F Fitzpatrick A Obesity, asthma, and oxidative stress J Appl Physiol 2010 108 754 759 10.1152/japplphysiol.00702.2009 19926826

[B42] Porter M Karp M Killedar S Bauer SM Guo J Williams D Breysse P Georas SN Williams MA Diesel-enriched particulate matter functionally activates human dendritic cells Am J Respir Cell Mol Biol 2007 37 706 719 10.1165/rcmb.2007-0199OC 17630318 PMC2219549

[B43] Chan RC Wang M Li N Yanagawa Y Onoé K Lee JJ Nel AE Pro-oxidative diesel exhaust particle chemicals inhibit LPS-induced dendritic cell responses involved in T-helper differentiation J Allergy Clin Immunol 2006 118 455 465 10.1016/j.jaci.2006.06.006 16890772

[B44] Yu KO Grabinski CM Schrand AM Murdock RC Wang W Gu B Schlager JJ Hussain SM Toxicity of amorphous silica nanoparticles in mouse keratinocytes J Nanopart Res 2008 11 15 24

[B45] Lin W Huang Y-W Zhou X-D Ma Y *In vitro* toxicity of silica nanoparticles in human lung cancer cells Toxicol Appl Pharmacol 2006 217 252 259 10.1016/j.taap.2006.10.004 17112558

[B46] Wang Y-H Wills-Karp M The potential role of interleukin-17 in severe asthma Curr Allergy Asthma Rep 2011 11 388 394 10.1007/s11882-011-0210-y 21773747 PMC4115366

[B47] Ather JL Ckless K Martin R Foley KL Suratt BT Boyson JE Fitzgerald KA Flavell RA Eisenbarth SC Poynter ME Serum amyloid A activates the NLRP3 inflammasome and promotes Th17 allergic asthma in mice J Immunol 2011 187 64 73 10.4049/jimmunol.1100500 21622869 PMC3119761

[B48] Besnard A-G Guillou N Tschopp J Erard F Couillin I Iwakura Y Quesniaux V Ryffel B Togbe D NLRP3 inflammasome is required in murine asthma in the absence of aluminum adjuvant Allergy 2011 66 1047 1057 10.1111/j.1398-9995.2011.02586.x 21443539

[B49] Zhou R Yazdi AS Menu P Tschopp J A role for mitochondria in NLRP3 inflammasome activation Nature 2011 469 221 225 10.1038/nature09663 21124315

[B50] Eisenbarth SC Colegio OR O’Connor W Sutterwala FS Flavell RA Crucial role for the Nalp3 inflammasome in the immunostimulatory properties of aluminium adjuvants Nature 2008 453 1122 1126 10.1038/nature06939 18496530 PMC4804622

[B51] Yazdi AS Guarda G Riteau N Drexler SK Tardivel A Couillin I Tschopp J Nanoparticles activate the NLR pyrin domain containing 3 (Nlrp3) inflammasome and cause pulmonary inflammation through release of IL-1α and IL-1β Proc Natl Acad Sci USA 2010 107 19449 19454 10.1073/pnas.1008155107 20974980 PMC2984140

[B52] Hansen G Berry G DeKruyff RH Umetsu DT Allergen-specific Th1 cells fail to counterbalance Th2 cell-induced airway hyperreactivity but cause severe airway inflammation J Clin Invest 1999 103 175 183 10.1172/JCI5155 9916129 PMC407883

[B53] Palomäki J Karisola P Pylkkänen L Savolainen K Alenius H Engineered nanomaterials cause cytotoxicity and activation on mouse antigen presenting cells Toxicology 2010 267 125 131 10.1016/j.tox.2009.10.034 19897006

[B54] Kadowaki N Antonenko S Lau JY Liu YJ Natural interferon alpha/beta-producing cells link innate and adaptive immunity J Exp Med 2000 192 219 226 10.1084/jem.192.2.219 10899908 PMC2193254

[B55] Shiow LR Rosen DB Brdicková N Xu Y An J Lanier LL Cyster JG Matloubian M CD69 acts downstream of interferon-alpha/beta to inhibit S1P1 and lymphocyte egress from lymphoid organs Nature 2006 440 540 544 10.1038/nature04606 16525420

[B56] Fonteneau J-F Gilliet M Larsson M Dasilva I Münz C Liu Y-J Bhardwaj N Activation of influenza virus-specific CD4+ and CD8+ T cells: a new role for plasmacytoid dendritic cells in adaptive immunity Blood 2003 101 3520 3526 10.1182/blood-2002-10-3063 12511409

[B57] Gao Y Majchrzak-Kita B Fish EN Gommerman JL Dynamic accumulation of plasmacytoid dendritic cells in lymph nodes is regulated by interferon-beta Blood 2009 114 2623 2631 10.1182/blood-2008-10-183301 19652204

[B58] Careau E Proulx L-I Pouliot P Spahr A Turmel V Bissonnette EY Antigen sensitization modulates alveolar macrophage functions in an asthma model Am J Physiol Lung Cell Mol Physiol 2006 290 L871 L879 10.1152/ajplung.00219.2005 16603596

[B59] Herbert C Scott MM Scruton KH Keogh RP Yuan KC Hsu K Siegle JS Tedla N Foster PS Kumar RK Alveolar macrophages stimulate enhanced cytokine production by pulmonary CD4+ T-lymphocytes in an exacerbation of murine chronic asthma Am J Pathol 2010 177 1657 1664 10.2353/ajpath.2010.100019 20724599 PMC2947263

[B60] Koike E Yanagisawa R Sadakane K Inoue K-I Ichinose T Takano H Effects of diisononyl phthalate on atopic dermatitis *in vivo* and immunologic responses *in vitro* Environ Health Perspect 2010 118 472 478 10.1289/ehp.118-a472 20064775 PMC2854722

[B61] Lacher SE Johnson C Jessop F Holian A Migliaccio CT Murine pulmonary inflammation model: a comparative study of anesthesia and instillation methods Inhal Toxicol 2010 22 77 83 10.3109/08958370902929969 20017595 PMC4068398

[B62] Beaudry C Lavoué J Sauvé J-F Bégin D Senhaji Rhazi M Perrault G Dion C Gérin M Occupational exposure to silica in construction workers: a literature-based exposure database J Occup Environ Hyg 2013 10 71 77 10.1080/15459624.2012.747399 23252413

[B63] Binauld S Boisson F Hamaide T Pascault J Drockenmuller E Fleury E Lyon D De Lyon I Lmm IMP Kinetic study of copper (I) -catalyzed click chemistry step-growth polymerization J Polym Sci, Part A: Polym Chem 2008 46 5506 5517 10.1002/pola.22871

[B64] Kitto HJ Schwartz E Nijemeisland M Koepf M Cornelissen JJLM Rowan AE Nolte RJM Post-modification of helical dipeptido polyisocyanides using the “click” reaction J Mater Chem 2008 18 5615 5624 10.1039/b811002f

[B65] Kar M Vijayakumar PS Prasad BLV Sen Gupta S Synthesis and characterization of poly-L-lysine-grafted silica nanoparticles synthesized via NCA polymerization and click chemistry Langmuir 2010 26 5772 5781 10.1021/la903595x 20337478

[B66] Delayre-Orthez C De Blay F Frossard N Pons F Dose-dependent effects of endotoxins on allergen sensitization and challenge in the mouse Clin Exp Allergy 2004 34 1789 1795 10.1111/j.1365-2222.2004.02082.x 15544606

[B67] Harkema JR Hotchkiss JA *In vivo* effects of endotoxin on intraepithelial mucosubstances in rat pulmonary airways. Quantitative histochemistry Am J Pathol 1992 141 307 317 1497089 PMC1886614

[B68] Harkema JR Plopper CG Hyde DM St George JA Regional differences in quantities of histochemically detectable mucosubstances in nasal, paranasal, and nasopharyngeal epithelium of the bonnet monkey J Histochem Cytochem 1987 35 279 286 10.1177/35.3.2434556 2434556

