# Supplementary material for: Engineered silica nanoparticles act as adjuvants to enhance allergic airway disease in mice
Source: Part Fibre Toxicol. 2013 Jul 1;10:26. doi: 10.1186/1743-8977-10-26 (PMC3729411; doi:10.1186/1743-8977-10-26)
Supplement: Additional file 1 — Additional information on material and methods. Further information on the design of engineered silica nanoparticles and on the gene expression analysis of the lung tissue are provided in the Additional file 1, as well as more detailed information on the gating strategy for FACS. [file 1743-8977-10-26-S1.pdf]

Engineered Silica Nanoparticles Act as Adjuvants to Enhance Allergic Airway Disease in Mice

Brandenberger C, Rowley NL, Jackson-Humbles DN, Zhang Q, Bramble LA, Lewandowski RP, Wagner JG, Chen W, Kaplan BL, Kaminski NE, Baker GL, Worden RM, Harkema JR

Content:

1. Design of Engineered Silica Nanoparticles
2. Supplementary Information Gene Expression Analysis
3. Gating strategy for FACS

1. Design of Engineered Silica Nanoparticles

Materials

Plain SNP (LUDOX® TM-40 colloidal silica, 40 wt% suspension in H_2O), aminopropyltriethoxysilane (APTES), propargyl chloroformate and sodium azide were purchased from Sigma-Aldrich and used as received. Anhydrous dimethylformamide (DMF) was freshly distilled from powered BaO. $Cu(PPh_3)Br$ [1] and 1-azido-2-(2-(2-(2-methoxyethoxy)ethoxy)ethoxyethane (PEG-N_3) [2] were prepared as previously described in detail. Other reagents and solvents were used as received from the supplier.

Instruments and characterization

^1H nuclear magnetic resonance (NMR) and ^{13}C NMR spectra were recorded in NMR solvent $CDCl_3$ on a Varian 300 MHz or VXR-500 MHz instrument. The $CDCl_3$ resonance was used as the internal standard for ^{13}C NMR (δ = 77.0 ppm) and residual $CHCl_3$ for ^1H NMR (δ = 7.24 ppm). Fourier transform infrared (FTIR) spectra were recorded on a Mattson Galaxy series FTIR 3000. Thermogravimetric analyses (TGA) were obtained in air from a Perkin-Elmer TGA 7. Samples were held at 120 °C for 30 min to remove absorbed water from particle surfaces and then heated to 850 °C at a rate of 10 °C/min. All samples for FTIR and TGA were vacuum dried at room temperature for 24 h.

Dynamic Light Scattering (DLS) was performed in a Malvern NanoZS ZetaSizer with a 178 degree backscattering detection. Intensity average diameters were calculated from the autocorrelation function using Malvern's Zetasizer Software 6.12. The samples for DLS analyses were sonicated prior to measuring the particle size (FS20H sonicator, Fischer Scientific). Centrifugation was performed at 6000 rpm on KμPrima-18R (Composite Rotor Inc.).

Surface Modification of Colloidal Silica Nanoparticles

As shown in figure S1, PEG-coated SNP were synthesized from plain SNP (commercially available LUDOX® TM-40 SNP) in three steps. To avoid aggregation, the modified SNP were purified by several steps of washing the nanoparticles with solvent, following by centrifugation. The intermediate particles (aSNP and aaSNP) were immediately further processed according the protocol and aggressive drying such as drying under vacuum [3] was avoided.

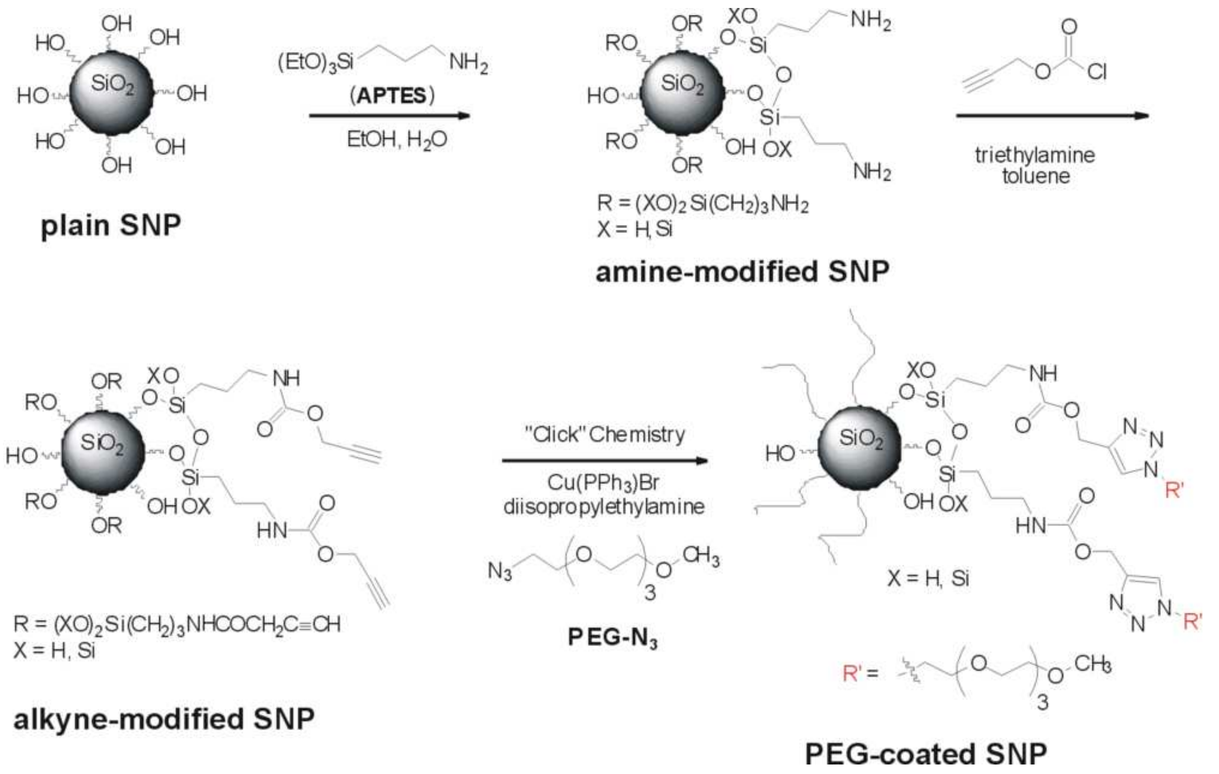

Figure S1. Process of SNP PEG coating. PEG-coated SNP were synthesized from commercially available LTM40 silica nanoparticles in three steps. First, aminopropyltriethoxy silane (APTES) was condensed on the plain silica particles, resulting amine-modified SNP (aSNP). Then, particles were reacted with propargyl chloroformate to afford the alkyne-modified particles (aaSNP). Finally, particles were clicked with PEG-N_3 catalyzed by 10% of Cu(PPh_3)Br diisopropylenamine to achieved PEG-coated SNP.

(1) Synthesis of amine-modified SNP (aSNP:. Commercially available plain SNP (LUDOX® TM-40 colloidal silica; 4.8 g in H_2O) were diluted with 100 mL of a 1:1 solution of EtOH/H_2O (v/v), and then aminopropyltriethoxy silane (APTES) (5 mL, 20 mmol) was drop wise added to the silica suspension. The mixture gradually evolved to a turpid suspension due to the formation of silane oligomers. After stirring for 3 days, the suspension was washed with EtOH and centrifuged (6000 rpm) for 20 - 40 min to remove the silane oligomer and any other impurities by. The purified product was kept in ethanol.

(2) Synthesis of alkyne-modified SNP (aaSNP): To remove ethanol and water, aSNP were washed and recovered by centrifugation (30 min) twice with reagent grade DMF, followed by washing three times with anhydrous DMF. The aSNP (30 mg, 1 equiv. of amine) were re-dispersed in a mixture of anhydrous DMF and triethylamine (3:1) by sonication (40 min) to obtain a homogenous solution. After cooling in an ice bath for 30 min, propargyl chloroformate (2 mL, 1000 equiv.) was added drop wise and the solution turned to slight yellow. After addition of propargyl chloroformate, the ice bath was removed and the mixture was stirred for 24 h. The product was purified by several cycles of washing with CH_2Cl_2 and recovery by centrifugation, until the solvent was colorless and no sign of residual amine salts. The product was stored in CH_2Cl_2 till further modification.

(3) Synthesis of PEG-coated SNP by "Click" modification of aaSN: estabished aaSNP (38 mg, 1 equiv. of alkyne) were dispersed in 50 mL of DMF, followed by sonication for 30 min. An excess of PEG-N_3 (3.4 equiv.) and 0.2 mL of N,N-diisopropylethylamine were added to the suspension, followed by three freeze-pump-thaw processes to remove O_2. Solid Cu(PPh_3)Br (10% equiv.) was added followed by a final freeze-pump-thaw process. After purging with N_2,

the suspension was stirred at RT for 24 h. The suspension was centrifuged to recover the PEGylated SNP (2x10 mL). The purified particles were kept in distilled water. The nanoparticle concentration was measured by drying fixed amounts of the aqueous solution.

Changes in the SNP surface chemistry were tracked by FTIR and figure S2 shows the normalized FTIR spectra of SNP after each modification step. Since the IR band at 806 cm^{-1} is characteristic for silica, IR data were normalized allowing semi-quantitative comparison of the nanoparticles during the syntheses. For example, the spectrum of aSNP shows increased absorption at 2900–3300 cm^{-1} and a new band at 1431 cm^{-1}, corresponding to the alkyl and amine groups of APTES. After treating aSNP with propargyl chloroformate, new absorption bands are seen at 1700 cm^{-1} due to C=O stretching, and triple bond stretching at 2127 cm^{-1}, confirming successful alkyne functionalization on the particle surface (aaSNP) [4]. IR is less useful for determining PEGylation; the characteristic C-O bands are obscured by the strong IR bands of the particles, but bands from C-H bending and stretching increased.

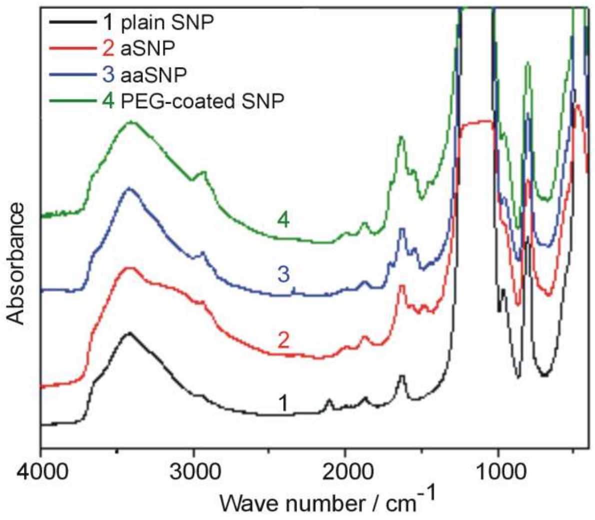

Figure S2. FTIR spectra of PEG-coated SNP and its precursors. The spectra are normalized by the absorbance at 888cm^{-1}, a band characteristic of SiO_2.

The TGA data in figure S3 and table 1 (main manuscript) confirm the step-by-step modification of silica particles. Since the TGA mass losses correspond to oxidation of the organic layer on the nanoparticles, the grafting density on the particle surface can be calculated. Table 1 in the article shows a 3.7% weight loss for amine-modified nanoparticles (aSNP), which corresponds to a grafting density of 2.7 group/nm^2. After the alkyne modification (aaSNP), the additional mass loss (2.4%) indicate that about half of the amines were converted to the triple bonds, and the pegylation step (3.6%) corresponds to a grafting density of 0.7 PEG chain/nm^2 and successful synthesis of water-soluble nanoparticles (PEG-coated SNP).

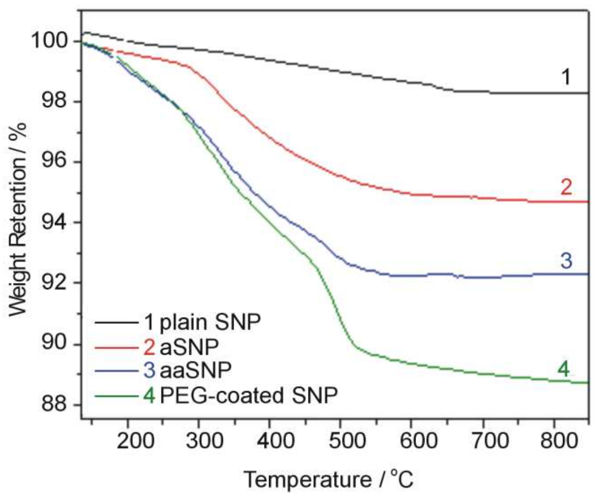

Figure S3. TGA data for PEG-coated SNP and its precursors. All samples were held at 120 °C for 30 min to remove absorbed water from particle surfaces, and then heated in air to 850 °C at a rate of 10 °C/min.

The DLS analysis shows that all nanoparticles have monomodal (Figure S4), but their size and size distribution depend on the surface chemistry of the particles, and the solvent used for the analysis. The average diameter of commercially available plain SNP is 30 nm (Table 1, article), showing a narrow particle distribution. Adding the amine to the particle surface increased the average diameter to 103 nm (DMF-NEt_3 1:3) and after alkyne modification (hydrophobic) the average particle size was 126 nm (DMF). PEGylation made the particles water soluble, the average diameter decreased to 90 nm and the distribution narrowed as indicated by figure S4 and table 1.

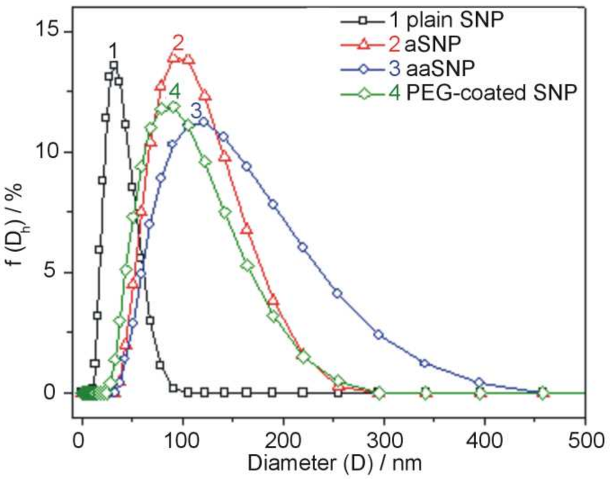

Figure S4. DLS data (size distribution by intensity) for PEG-coated SNP and its precursors.

References

1. Binauld S, Boisson F, Hamaide T, Pascault J, Drockenmuller E, Fleury E, Lyon D, Lyon ID, Lmm IMP: **Kinetic Study of Copper (I) -Catalyzed Click Chemistry Step-Growth Polymerization**. *J Polym Sci, Part A: Polym Chem* 2008, **46**:5506–5517.

2. Kitto HJ, Schwartz E, Nijemeisland M, Koepf M, Cornelissen JJLM, Rowan AE, Nolte RJM: **Post-modification of helical dipeptido polyisocyanides using the "click" reaction**. *J Mater Chem* 2008, **18**:5615–5624.

3. Kar M, Vijayakumar PS, Prasad BLV, Sen Gupta S: **Synthesis and characterization of poly-L-lysine-grafted silica nanoparticles synthesized via NCA polymerization and click chemistry.** *Langmuir* 2010, **26**:5772–5781.

4. Chandran SP, Hotha S, Prasad BLV: **Tunable surface modification of silica nanoparticles through " click " chemistry**. *Current Science* 2008, **95**:1327–1333.

2. Supplementary Information Gene Expression Analysis

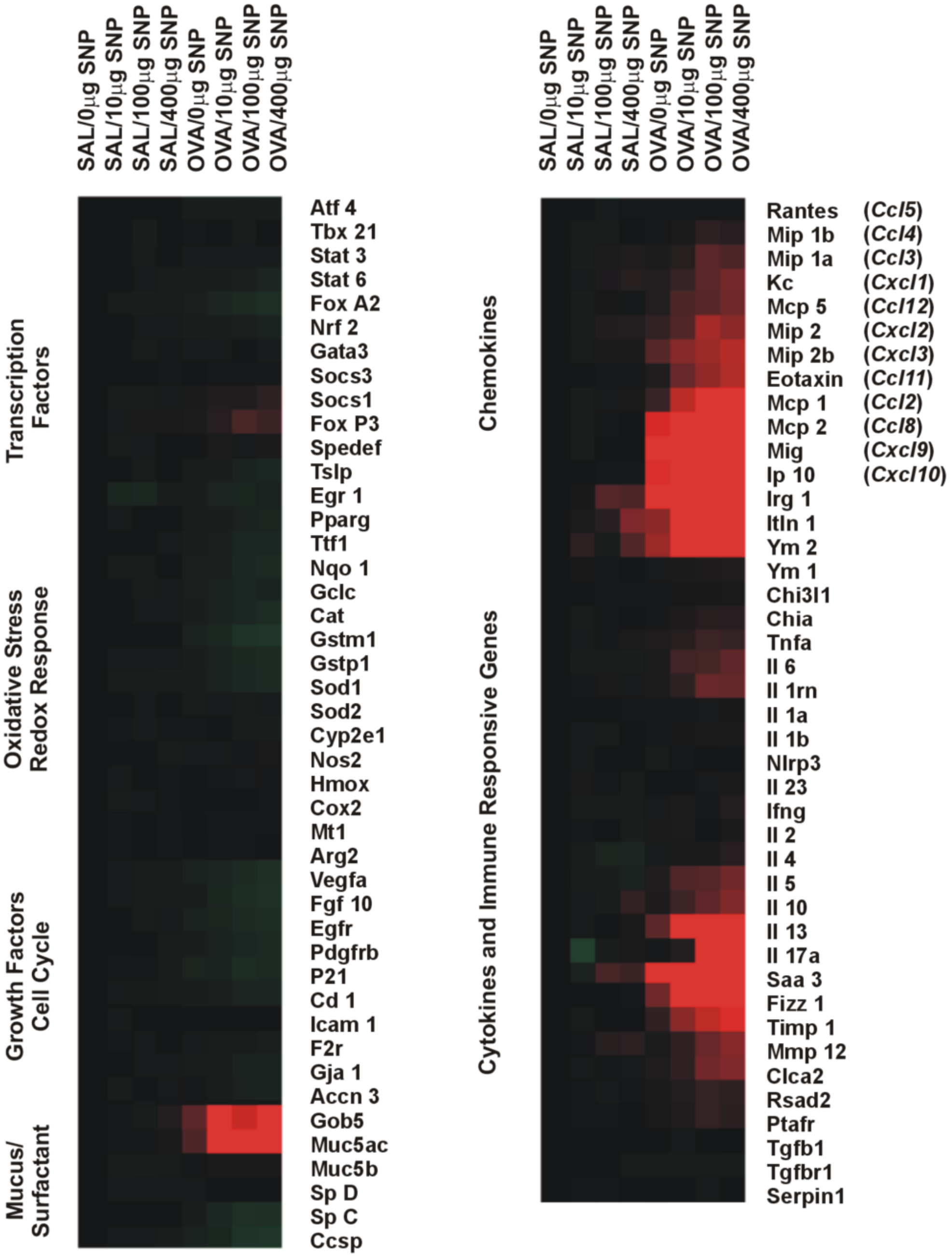

Figure S5. Heat map of gene expression array. Gene expression in lung tissue was analyzed with a 96-gene array. Relative increased gene expression towards control is indicated in red (≥2 fold expression) and relative decreased gene expression in green (≤2 fold). Black labels indicate no differences in gene expression. Increased gene expression in allergic and SNP exposed animals was found for various cytokines, chemokines and immune responsive genes as well as secretory mucus/surfactant genes. No changes greater than 2-fold were found for oxidative stress response, growth factors and different transcription factors.

Table S1. *Taq*Man Gene Expression analysis. Detailed list of all genes and primers used for gene expression analysis with the *Taq*Man Gene Expression Assay from Applied Biosystems.

Gene Symbol	Gene name	Gene Alias	Array Group	Assay ID	NCBI Gene Reference
18S	18S rRNA		Endogenous	Mm03928990_g1	NR_003286.1
Actb	Actin, beta		Endogenous	Mm00607939_s1	NM_001101.3
Arbp	Acidic ribosomal phosphoprotein P0	ARBP (Endog)	Endogenous	Mm01974474_gH	NM_203736.1
Gapd	Glyceraldehyde-3-phosphate dehydrogenase	GAPDH	Endogenous	Mm99999915_g1	NM_017008.3
Gusb	Glucuronidase, beta	Gus	Endogenous	Mm00446953_m1	NM_010368.1
Hprt1	Hypoxanthine guanine phosphoribosyl transferase	Hprt	Endogenous	Mm00446968_m1	NM_013556.2
Ccl2	Chemokine (C-C motif) ligand 2	MCP-1, Scya-2	Chemokine	Mm00441242_m1	NM_011333.3
Ccl3	Chemokine (C-C motif) ligand 3	MIP1-alpha	Chemokine	Mm00441258_m1	NM_011337.2
Ccl4	Chemokine (C-C motif) ligand 4	MIP1-beta	Chemokine	Mm00443111_m1	NM_013652.2
Ccl5	Chemokine (C-C motif) ligand 5	RANTES	Chemokine	Mm01302427_m1	NM_013653.3
Ccl8	Chemokine (C-C motif) ligand 8	MCP-2	Chemokine	Mm01297183_m1	NM_021443.2
Ccl11	Chemokine (C-C motif) ligand 11	Eotaxin, Scya11	Chemokine	Mm00441238_m1	NM_011330.3
Ccl12	Chemokine (C-C motif) ligand 12	MCP5	Chemokine	Mm01211783_g1	NM_011331.2
Cxcl1	Chemokine (C-X-C motif) ligand 1	KC/CINC-1/ /IL8	Chemokine	Mm00433859_m1	NM_008176.3
Cxcl2	Chemokine (C-X-C motif) ligand 2	MIP-2	Chemokine	Mm00436450_m1	NM_009140.2
Cxcl3	Chemokine (C-X-C motif) ligand 3	MIP-2b,Dcip1,	Chemokine	Mm01701838_m1	NM_203320.2
Cxcl9	Chemokine (C-X-C motif) ligand 9	CMK, MIG, crg10	Chemokine	Mm01345157_m1	NM_008599.4
Cxcl10	Chemokine (C-X-C motif) ligand 10	IP-10, Ifi10, gIP10	Chemokine	Mm00445235_m1	NM_021274.1
Ifng	Interferon gamma	IFN-g	Cytokine	Mm00801778_m1	NM_008337.3
Il1a	Interleukin 1 alpha		Cytokine	Mm00439620_m1	NM_010554.4
Il1b	Interleukin 1 beta		Cytokine	Mm00434228_m1	NM_008361.3
Il2	Interleukin 2		Cytokine	Mm00434256_m1	NM_008366.2
Il4	Interleukin 4		Cytokine	Mm00445259_m1	NM_021283.2
Il5	Interleukin 5		Cytokine	Mm00439646_m1	NM_010558.1
Il6	Interleukin 6		Cytokine	Mm00446190_m1	NM_031168.1
Il10	Interleukin 10		Cytokine	Mm00439616_m1	NM_010548.1
Il13	Interleukin 13		Cytokine	Mm00434204_m1	NM_008355.3
Il17a	Interleukin 17A	Ctla8	Cytokine	Mm00439618_m1	NM_010552.3
Il23a	Interleukin 23	p19	Cytokine	Mm00518984_m1	NM_031252.2
Tgfb1	Transforming growth factor, beta 1	TGF-beta1	Cytokine	Mm01178820_m1	NM_011577.1
Tnfa	Tumor necrosis factor alpha	TNF alpha	Cytokine	Mm00443258_m1	NM_013693.2
Il1rn	Interleukin 1 receptor antagonist		Cytokine receptor	Mm01337566_m1	NM_031167.5
TgfbR1	Transforming growth factor, beta receptor I	TbetaRI	Cytokine receptor	Mm00436971_m1	NM_009370.2
Timp1	Tissue inhibitor of metalloproteinase 1		Cytokine receptor	Mm00441818_m1	NM_001044384.1
Irg1	Immunoresponsive gene 1		Inflamatory	Mm01224529_m1	NM_008392.1
Itln1	Intelectin 1 (galactofuranose binding)	Itlna	Inflamatory	Mm00843942_sH	NM_010584.2
Nlrp3	NLR family, pyrin domain containing 3	FCAS, FCU	Inflamatory	Mm00840904_m1	NM_145827.3
Ptafr	Platelet-activating factor receptor	PAFR	Inflamatory	Mm02621061_m1	NM_001081211.1

Gene Symbol	Gene name	Gene Alias	Array Group	Assay ID	NCBI Gene Reference
Retnla	Resistin like alpha	Fizz-1	Inflamatory	Mm00445109_m1	NM_020509.3
Rsad2	Radical S-adenosyl methionine domain containing 2		Inflamatory	Mm00491265_m1	NM_021384.4
Saa3	Serum amyloid A3	Saa-3	Inflamatory	Mm00441203_m1	NM_011315.3
Serpin1	Serine peptidase inhibitor	PAI-1	Inflamatory	Mm01204469_m1	NM_008871.2
Tfpi2	Tissue factor pathway inhibitor 2		Inflamatory	Mm00436948_m1	NM_009364.3
Chi3l1	Chitinase 3-like 1	Brp39, Gp39	Chitinase	Mm00801477_m1	NM_007695.3
Chi3l3	Chitinase 3-like 3	YM1,ECF-L	Chitinase	Mm00657889_mH	NM_009892.2
Chi3l4	Chitinase 3-like 4	YM2	Chitinase	Mm00840870_m1	NM_145126.2
Chia	Chitinase, acidic	AMCase	Chitinase	Mm00458221_m1	NM_023186.3
Ccnd1	Cyclin D1	CD1,Cyl-1	Cell Cycle	Mm03053889_s1	NM_007631.2
Cdkn1a	Cyclin-dependent kinase inhibitor 1A	P21	Cell Cycle	Mm01303209_m1	NM_007669.4
Gas1	Growth arrest specific 1	Gas-1	Cell Cycle	Mm01700206_g1	NM_008086.1
Pcna	Proliferating cell nuclear antigen	Pcna	Cell Cycle	Mm00448100_g1	NM_011045.2
Fgf10	Fibroblast growth factor 10	FGF-10	Growth factor	Mm00433275_m1	NM_008002.4
Tff2	Trefoil factor 2 (spasmolytic protein 1)	SP, mSP	Growth factor	Mm00447491_m1	NM_009363.3
Vegfa	Vascular endothelial growth factor A	VPF, Vegf	Growth factor	Mm01281447_m1	NM_001025250.3
Egfr	Epidermal growth factor receptor	Egfr	Growth factor receptor	Mm00433023_m1	NM_207655.2
Pdgfrb	Platelet derived growth factor receptor, beta	Pdgfr	Growth factor receptor	Mm01262489_m1	NM_001146268.1
Accn3	Amiloride-sensitive cation channel 3	ASIC3	Ion channel	Mm00805460_m1	NM_183000.2
Clca2	Chloride channel calcium activated 2		Ion channel	Mm00661630_m1	NM_030601.2
Clca3	Chloride channel calcium activated 3	Gob-5	Ion channel	Mm00489959_m1	NM_017474.1
F2r	Coagulation factor II (thrombin) receptor	Par1, ThrR	Coagulation	Mm00438851_m1	NM_010169.3
Gja1	Gap junction protein, alpha 1		Cell adhesion	Mm00439105_m1	NM_010288.3
Icam1	Intercellular adhesion molecule 1		Cell adhesion	Mm00516023_m1	NM_010493.2
Arg2	Arginase type II		Oxidoreductase	Mm00477592_m1	NM_009705.3
Cat	Catalase	Cas1,Cs-1	Oxidoreductase	Mm00437992_m1	NM_009804.2
Cyp2e1	Cytochrome P450, family 2e1		Oxidoreductase	Mm00491127_m1	NM_021282.2
Mt1	Metallothionein 1		Oxidoreductase	Mm00496660_g1	NM_013602.3
Nqo1	NAD(P)H dehydrogenase, quinone 1	Nmo-1, Ox-1	Oxidoreductase	Mm00500821_m1	NM_008706.5
Sod1	Superoxide dismutase 1, soluble		Oxidoreductase	Mm01344233_g1	NM_011434.1
Sod2	Superoxide dismutase 2, mitochondrial	MnSOD	Oxidoreductase	Mm00449726_m1	NM_013671.3
Hmox	Heme oxygenase (decycling) 1	HO-1	Oxidoreductase	Mm00516004_m1	NM_010442.2
Mmp12	Matrix metallopeptidase 12		Protease	Mm00500554_m1	NM_008605.3
Muc5ac	Mucin 5, subtypes A & C		Secretory	Mm01276725_g1	NM_010844.1
Muc5b	Mucin 5, subtype B		Secretory	Mm00466376_m1	NM_028801.2
Scgb1a 1	Clara cell secretory protein	CCSP, CC10	Secretory	Mm00442046_m1	NM_011681.2
Sftpc	Surfactant protein C	SP-C	Surfactant	Mm00488144_m1	NM_011359.1
Sftpd	Surfactant protein D	SP-D	Surfactant	Mm00486060_m1	NM_009160.2
Nos2	Nitric oxide synthase 2, inducible	iNOS	Synthase	Mm00440485_m1	NM_010927.3
Ptgs2	Prostaglandin-endoperoxide synthase 2	COX2	Synthase	Mm00478374_m1	NM_011198.3
Gclc	Glutamate-cysteine ligase, catalytic subunit		Synthase	Mm00802655_m1	NM_010295.1
Gstm1	Glutathione S-transferase, mu 1	Gstb1	Transferase	Mm00833915_g1	NM_010358.5

Gene Symbol	Gene name	Gene Alias	Array Group	Assay ID	NCBI Gene Reference
Gstp1	Glutathione S-transferase, pi 1	GstpiB	Transferase	Mm00496606_m1	NM_013541.1
Egr1	Early growth response 1	Egr	Transcription factor	Mm00656724_m1	NM_007913.5
Gata3	GATA binding protein 3		Transcription factor	Mm00484683_m1	NM_008091.3
Spdef	SAM pointed domain containing ets transcription factor	PDEF, Pse	Transcription factor	Mm00600221_m1	NM_013891.4
Stat3	Signal transducer and activator of transcription 3	Aprf	Transcription factor	Mm01219775_m1	NM_213659.2
Stat6	Signal transducer and activator of transcription 6		Transcription factor	Mm01160477_m1	NM_009284.2
Tbx21	T-box 21	T-bet, TBT1	Transcription factor	Mm00450960_m1	NM_019507.2
Tslp	Thymic stromal lymphopoietin		Transcription factor	Mm00498739_m1	NM_021367.1
Ttf1	Transcription termination factor, RNA polymerase I		Transcription factor	Mm00657017_m1	NM_009442.2
Atf4	Activating transcription factor 4		Transcription factor	Mm00515324_m1	NM_009716.2
Foxa2	Forkhead box A2	HNF3-beta	Transcription factor	Mm00839704_mH	NM_010446.2
Foxp3	Forkhead box P3	JM2	Transcription factor	Mm00475165_m1	NM_054039.1
Nfe2l2	Nuclear factor, erythroid derived 2, like 2	Nrf2	Transcription factor	Mm00477784_m1	NM_010902.3
Pparg	Peroxisome proliferator activated receptor gamma	PPAR-g	Transcription factor	Mm00440945_m1	NM_001127330.1
Socs1	Suppressor of cytokine signaling 1	SSI-1	Transcription factor	Mm00782550_s1	NM_009896.2
Socs3	Suppressor of cytokine signaling 3	SOC3	Transcription factor	Mm00545913_s1	NM_007707.2

3. Gating Strategy for FACS

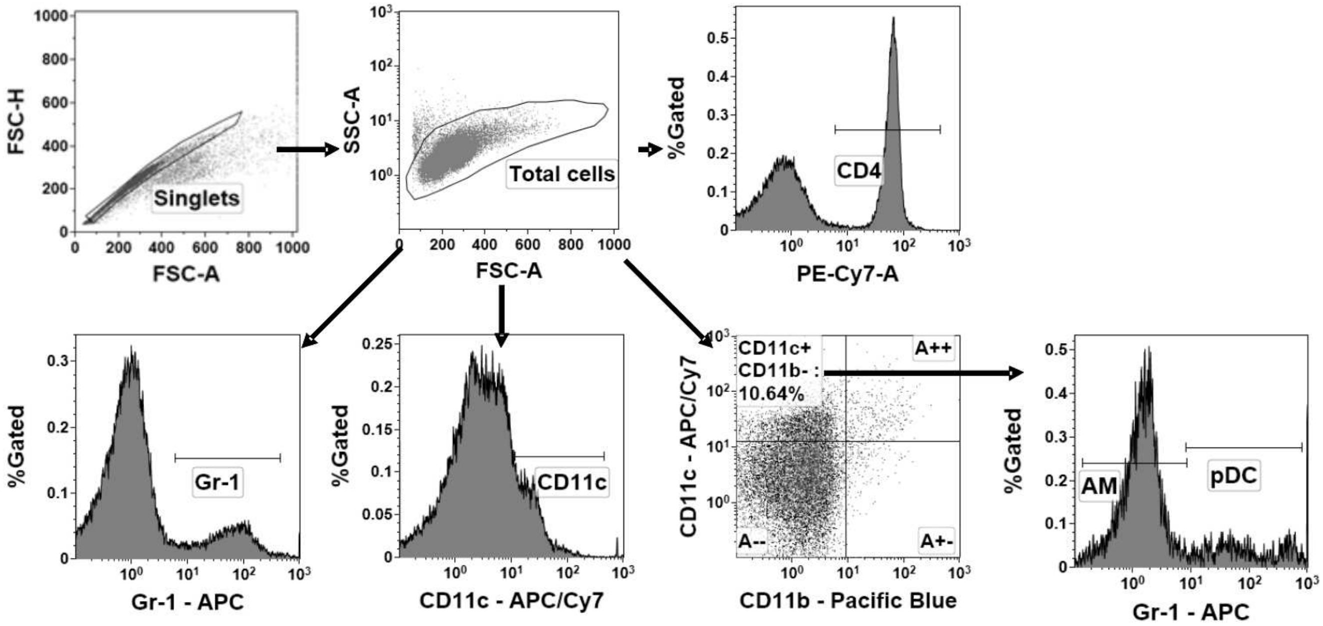

Figure S6. Cells from TBLN were stained for expression of surface molecules, including CD4, CD69, Cd11c, CD11b, MHC II, and Gr-1. Cells were first gated on singlets using FSC-A/FSC-H, then gated on total cells, including lymphocytes, monocytes, and granulocytes, using FSC-A/SSC-A. Subsequently, the total cell population was gated on $CD4^+$, Gr-1^+, $CD11c^+$, or $CD11c^+CD11b^-$ population, respectively. The $CD11c^+CD11b^-$ population was further gated on Gr-1^+ for AM and Gr-1^- for pDC.
